# Adeno-associated viral vectors for functional intravenous gene transfer throughout the non-human primate brain

**DOI:** 10.1038/s41565-023-01419-x

**Published:** 2023-07-10

**Authors:** Miguel R. Chuapoco, Nicholas C. Flytzanis, Nick Goeden, J. Christopher Octeau, Kristina M. Roxas, Ken Y. Chan, Jon Scherrer, Janet Winchester, Roy J. Blackburn, Lillian J. Campos, Kwun Nok Mimi Man, Junqing Sun, Xinhong Chen, Arthur Lefevre, Vikram Pal Singh, Cynthia M. Arokiaraj, Timothy F. Shay, Julia Vendemiatti, Min J. Jang, John K. Mich, Yemeserach Bishaw, Bryan B. Gore, Victoria Omstead, Naz Taskin, Natalie Weed, Boaz P. Levi, Jonathan T. Ting, Cory T. Miller, Benjamin E. Deverman, James Pickel, Lin Tian, Andrew S. Fox, Viviana Gradinaru

**Affiliations:** 1 Division of Biology and Biological Engineering, California Institute of Technology, Pasadena, CA USA; 2 Aligning Science Across Parkinson’s (ASAP) Collaborative Research Network, Chevy Chase, MD USA; 3 Department of Psychology and the California National Primate Research Center, University of California Davis, Davis, CA USA; 4 Department of Biochemistry and Molecular Medicine, University of California Davis, Davis, CA USA; 5 Cortical Systems and Behavior Laboratory, University of California San Diego, San Diego, CA USA; 6 Allen Institute for Brain Science, Seattle, WA USA; 7 Washington National Primate Research Center, University of Washington, Seattle, WA USA; 8 National Institute of Mental Health, National Institutes of Health, Bethesda, MD USA; 9 Present Address: Capsida Biotherapeutics, Thousand Oaks, CA USA; 10 Present Address: Stanley Center for Psychiatric Research at Broad Institute of MIT and Harvard, Massachusetts Institute of Technology, Cambridge, MA USA

**Keywords:** Biotechnology, Nanobiotechnology, Biosensors

## Abstract

Crossing the blood–brain barrier in primates is a major obstacle for gene delivery to the brain. Adeno-associated viruses (AAVs) promise robust, non-invasive gene delivery from the bloodstream to the brain. However, unlike in rodents, few neurotropic AAVs efficiently cross the blood–brain barrier in non-human primates. Here we report on AAV.CAP-Mac, an engineered variant identified by screening in adult marmosets and newborn macaques, which has improved delivery efficiency in the brains of multiple non-human primate species: marmoset, rhesus macaque and green monkey. CAP-Mac is neuron biased in infant Old World primates, exhibits broad tropism in adult rhesus macaques and is vasculature biased in adult marmosets. We demonstrate applications of a single, intravenous dose of CAP-Mac to deliver functional GCaMP for ex vivo calcium imaging across multiple brain areas, or a cocktail of fluorescent reporters for Brainbow-like labelling throughout the macaque brain, circumventing the need for germline manipulations in Old World primates. As such, CAP-Mac is shown to have potential for non-invasive systemic gene transfer in the brains of non-human primates.

## Abstract (web summary)

Crossing the blood–brain barrier in primates is a major obstacle to gene delivery in the brain. Here an adeno-associated virus variant (AAV.CAP-Mac) is identified and demonstrated for crossing the blood–brain barrier and delivering gene sequences to the brain of different non-human primates species.

## Main

Adeno-associated viruses (AAVs) were first identified as adenoviral contaminants in the 1960s^1–3^, and hundreds of clinical trials have since established their potential for the long-term expression of genetic payloads^4–7^. There is, however, renewed concern about the safety of high-dose systemic AAV delivery following reports of adverse hepatotoxicity^8,9^ and several patient deaths^10,11^. Natural AAV serotypes are characterized by a low therapeutic index and high effective dose, and as a result, there is an urgent need for more efficient—and thus safer—AAVs, particularly for the brain.

In recent years, the gene therapy field has focused on engineering novel capsids to address this problem and expand the window of therapeutic opportunity of AAVs. In parallel, the neuroscience community has engineered several AAV variants that can traverse the restrictive blood–brain barrier (BBB). Some of the first variants to efficiently traverse the BBB after intravenous (IV) administration in mice (AAV-PHP.B/eB) were engineered using Cre-recombination-based AAV targeted evolution (CREATE), which leverages Cre-transgenic mouse lines to impose additional selective pressure during library selections^12,13^. Further engineering efforts have since produced variants with enhanced neurotropic properties, such as the ability to cross the BBB across mouse strains, decreased transduction outside of the central nervous system (CNS), and biased tropism towards specific cell types^13–18^. With these advances, AAVs are now routinely used to systemically deliver genetically encoded tools—such as GCaMP to detect intracellular calcium gradients—to the mouse brain^12,13,15,16,19^.

By contrast, tools for gene transfer from the bloodstream to the CNS of non-human primates (NHPs) are scarce. Some capsids selected in rodents translate to the marmoset (*Callithrix jacchus*)^15^, a New World primate species, but few translate to Old World primates (OWPs), which are more evolutionarily related to humans and are well-established models of human cognition, neurodevelopment, neuroanatomy and physiology^20–23^. In particular, despite its success in mice, AAV-PHP.B does not cross the BBB in the rhesus macaque (*Macaca mulatta*)^24,25^, an OWP. In lieu of a BBB-penetrating vector for OWPs, researchers and clinicians resort to direct intraparenchymal injections, which, although effective, are intractable for brain-wide gene transfer. Several groups have attempted to circumvent the BBB via lumbar puncture^26^ or intra-cisterna magna (ICM)^27^ injections to access the cerebral spinal fluid. However, these routes of administration have limited efficacy in the brain^27–31^, and there are reports of adverse pathology in non-brain tissue, especially in the dorsal root ganglia^9,31–33^. To enable novel research in NHP animal models and for greater therapeutic translatability, it is imperative to advance AAV development for systemic gene transfer to the brains of OWPs such as macaques.

**Fig. 1:**
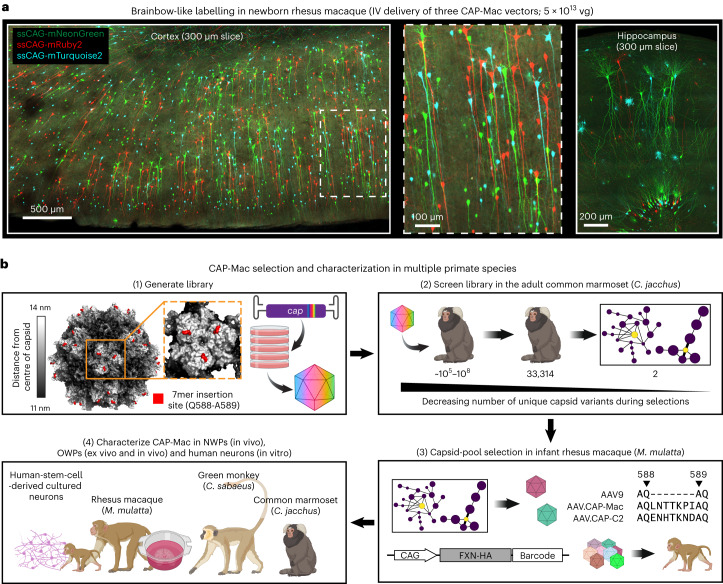
CAP-Mac selection and characterization strategy. **a**, AAV.CAP-Mac is a novel vector that enables brain-wide, systemic gene transfer in NHPs. Representative images are shown from a newborn rhesus macaque brain expressing three fluorescent reporters delivered intravenously using AAV.CAP-Mac (total dose 5 × 10^13^ vg, 4 weeks post-injection). **b**, Schematic of the CAP-Mac selection strategy. (1) CAP-Mac is an AAV9 variant that we selected from a library screened in the adult common marmoset. We generated diversity by introducing 7 NNK degenerate codons after Q588 in the AAV9 *cap* genome and produced the capsid library for in vivo selections in adult male marmosets. (2) In two rounds of selections, we intravenously administered 2 × 10^12^ vg per marmoset (two marmosets per round), narrowing our variant pool with each round of selection. After the first round of selection, we recovered 33,314 unique amino acid sequences in the brain. For the second round of selection, we generated a synthetic oligo pool containing each unique variant plus a codon-modified replicate (66,628 total sequences). After the second round of selection, we constructed network graphs of high-performing variants, and selected two capsids—AAV.CAP-Mac and AAV.CAP-C2—to be included in the pool selections in newborn rhesus macaques. (3) For pool selections, we produced eight capsids packaging ssCAG-hFXN-HA, each with a unique molecular barcode in the 3′ UTR. This construct design enabled us to assess the protein expression of the pool by staining for the HA epitope and quantify the barcodes in viral DNA and whole RNA extracts. We injected 1 × 10^14^ vg kg^−^^1^ of the virus pool into two newborn rhesus macaques via the saphenous vein and recovered tissue 4 weeks post-injection. (4) We moved forward with the individual characterization of AAV.CAP-Mac in various contexts (ex vivo, in vitro and in vivo) in multiple primate species.

Here we describe AAV.CAP-Mac, an engineered AAV9 variant that efficiently targets the CNS in both New World primates and OWPs. CAP-Mac is biased towards neurons in infant OWPs and vasculature in adult marmosets (cell-type tropisms missing in currently available AAVs) and demonstrates substantial improvement over AAV9 in adult macaque tissue (ex vivo and in vivo). CAP-Mac efficiently transduces neurons in the brains of at least two infant OWP species, namely, rhesus macaques and green monkeys (*Chlorocebus sabaeus*), targeting neurons in the CNS more effectively than its parent AAV9. To demonstrate CAP-Mac’s immediate research utility, we capitalized on its neuronal bias to express (1) functional GCaMP for ex vivo two-photon calcium imaging and (2) a cocktail of fluorescent reporters for Brainbow-like^34,35^ multicolour labelling and morphological tracing in the macaque brain (Fig. 1a). By characterizing CAP-Mac in multiple NHP species, we aim to both expand the AAV toolbox available to researchers interested in studying the OWP CNS and highlight the utility of engineering AAVs for increased translatability in higher-order mammals.

## Selecting brain-enriched AAVs using multiple NHP species

Our overarching goal was to develop an AAV variant efficacious in NHPs after systemic administration. To do that, we used a multispecies screening and characterization strategy to select for variants with enhanced BBB-crossing tropism in NHPs (Fig. 1b and Supplementary Fig. 1a–c). We initially screened a library of variants in the adult marmoset, where we identified 33,314 variants in the brain.

In the past, we used our CREATE methodology to increase stringency during selections by recovering only variants that underwent cis-Cre-Lox-mediated inversion^12,16^. However, since Cre-transgenic marmosets are still being developed and not yet available^36^, we pursued other strategies to compensate for the lack of this additional selective pressure. Previously, we clustered capsid variants based on sequence similarity to aid in choosing variants for further characterization^16^. We applied a similar analysis to our next-generation sequencing (NGS) datasets (Supplementary Fig. 1d–g), reasoning that this would let us limit the number of NHPs used and compensate for the absence of the CREATE selective pressure. Based on these network graphs, we chose two variants from the marmoset selection for further characterization: AAV.CAP-Mac (CAP-Mac) and AAV.CAP-C2 (CAP-C2).

**Fig. 2:**
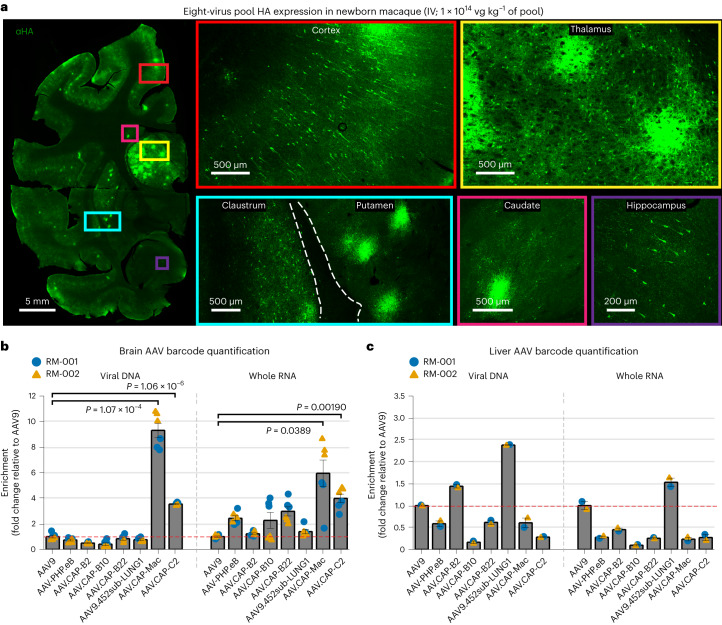
CAP-Mac outperforms other engineered variants in newborn rhesus macaque in pool testing. **a**, Representative images of the expression in cortex, thalamus, caudate nucleus, putamen, hippocampus and claustrum after IV administration of 1 × 10^14^ vg kg^−^^1^ of an eight-capsid pool (1.25 × 10^13^ vg kg^−^^1^ of each variant) packaging HA-tagged human frataxin with a unique barcode in each capsid. HA epitope expression in the cortex and hippocampus is observable in single cells with clear projections that resemble the apical dendrites of pyramidal cells. Furthermore, the thalamus and dorsal striatum contain areas of dense HA expression relative to other brain regions. The white dashed line signifies the difference between ‘viral DNA’ and ‘whole RNA’ sections of the plot. **b**,**c**, Unique barcode enrichments in viral DNA (left) and whole RNA (right) extracts from the brain (**b**; *n* = 6 brain samples from two newborn macaques) and the liver (**c**; *n* = 2 liver tissue samples from two newborn macaques). Each data point represents the fold change relative to AAV9 within each tissue sample. Mean values ± standard error of the mean (s.e.m.) are shown. The red dashed line denotes AAV9 performance in the pool. One-way analysis of variance using Tamhane’s T2 correction was tested against AAV9 enrichment. Source data

Following the marmoset selection, we used capsid-pool studies in newborn rhesus macaques to assess translatability to OWPs. We pooled eight capsid variants: AAV9, CAP-Mac, CAP-C2 and five previously engineered AAVs^13,15,37^. Each variant packaged a single-stranded human frataxin transgene fused to a hemagglutinin (HA) epitope tag under the control of a ubiquitous CAG promoter (ssCAG-hFXN-HA) with a unique molecular barcode in the 3′ UTR (Fig. 1b). After injecting the pool into newborn macaques, we observed robust HA epitope expression throughout the brain, confirming efficient transgene delivery mediated by one or more variants in the capsid pool (Fig. 2a). We quantified the relative enrichment of each barcode in the brain and found that the CAP-Mac-delivered barcode was nine and six times more abundant than the AAV9-delivered barcode in the viral DNA and total RNA, respectively (Fig. 2b). The CAP-C2-delivered barcode was approximately fourfold enriched relative to the AAV9 barcode in both DNA and RNA extracts. Interestingly, the viral DNA levels of all the other variants, which were originally selected in mice, were on par with AAV9, including AAV.CAP-B10 and AAV.CAP-B22, which cross the BBB in marmosets^15^. In the liver, CAP-Mac and CAP-C2 were negatively enriched (Fig. 2c), as were some of the previously engineered controls known to be de-targeted from the liver in rodents^15^ (that is, AAV.CAP-B10 and AAV.CAP-B22).

## CAP-Mac efficiently transduces neurons in infant OWP CNS

**Fig. 3:**
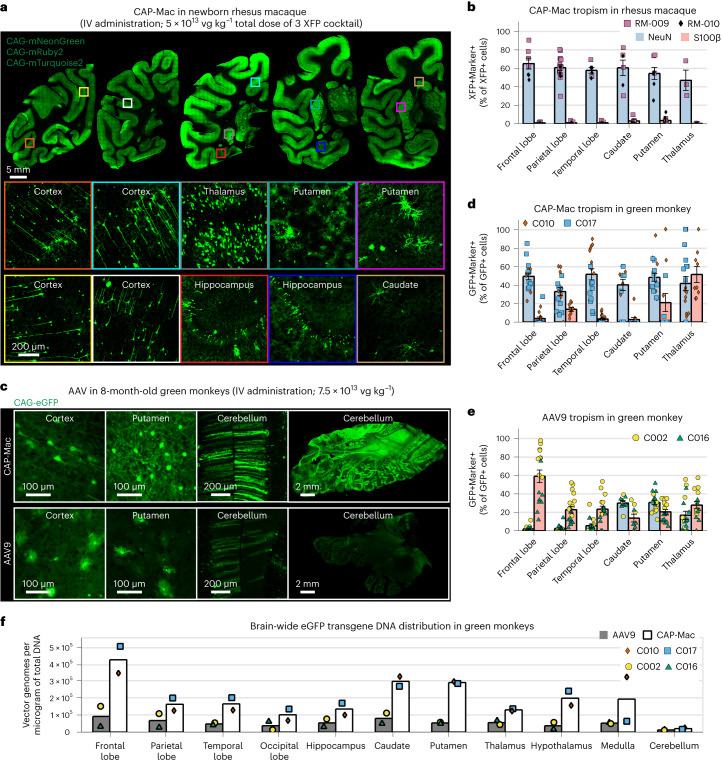
CAP-Mac is biased towards neurons throughout infant green monkey and newborn rhesus macaque brains. **a**, Distribution of IV CAP-Mac expression in 2-day-old rhesus macaques (5 × 10^13^ vg kg^−^^1^ via the saphenous vein) across coronal slices showing fluorescent reporter expression (ssCAG-mNeonGreen, ssCAG-mRuby2 and ssCAG-mTurquoise2) in the cortical and subcortical brain regions (insets). Imaging channels of reporters are identically pseudo-coloured. **b**, Co-localization of fluorescent reporters with NeuN (neurons) or S100β (astrocytes) in 2-day-old rhesus macaques treated with CAP-Mac (*n* = 2 macaques). Values are reported as a percentage of all the XFP+ cells. Mean [XFP+NeuN+]/XFP+ range between 47% and 60% and mean [XFP + S100β+]/XFP+ between 0% and 3%. Overall, CAP-Mac targeted 1.12% and 0.04% of all NeuN+ and S100β+ cells, respectively. **c**, Representative images from 8-month-old green monkeys intravenously dosed with CAP-Mac (top) or AAV9 (bottom) packaging ssCAG-eGFP (7.5 × 10^13^ vg kg^−^^1^ via the saphenous vein). **d**,**e**, Co-localization of fluorescent reporters with NeuN (neurons) or S100β (astrocytes) in infant green monkeys treated with CAP-Mac (**d**; *n* = 2 green monkeys) or AAV9 (**e**; *n* = 2 green monkeys). Values are reported as a percentage of all the GFP+ cells. CAP-Mac, mean [GFP+NeuN+]/GFP+ between 33% and 51% and mean [GFP+S100β+]/GFP+ between 3% and 21%. AAV9, mean [GFP+NeuN+]/GFP+ between 2% and 10% and mean [GFP+S100β+]/GFP+ between 23% and 59%. Overall, CAP-Mac targeted 1.30% and 0.64% of NeuN+ and S100β+ cells, respectively, in the green monkey brain. In contrast, AAV9 targeted 0.49% of NeuN+ cells and 1.86% of S100β+ cells. **f**, Distribution of CAP-Mac- and AAV9-delivered eGFP transgene in 11 brain regions of green monkeys (*n* = 4 green monkeys). Each data point represents the measured vector genomes per microgram of total DNA in a section of tissue from each region and monkey. Mean values ± s.e.m. are shown. Source data

**Extended Data Fig. 1:**
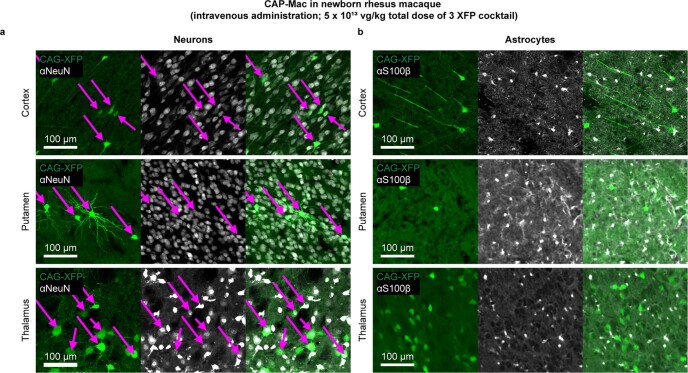
CAG-XFP co-localization with cell-type-specific histological markers. **a**, **b**, Representative images of a cocktail of 3 fluorescent proteins under control of CAG in newborn rhesus macaque tissue with histological markers for neurons (NeuN, **a**) and astrocytes (S100β, **b**). Cells that are positive for both fluorescent protein and histological marker are shown with a purple arrow. Fluorescent proteins are identically pseudo-coloured.

**Extended Data Fig. 2:**
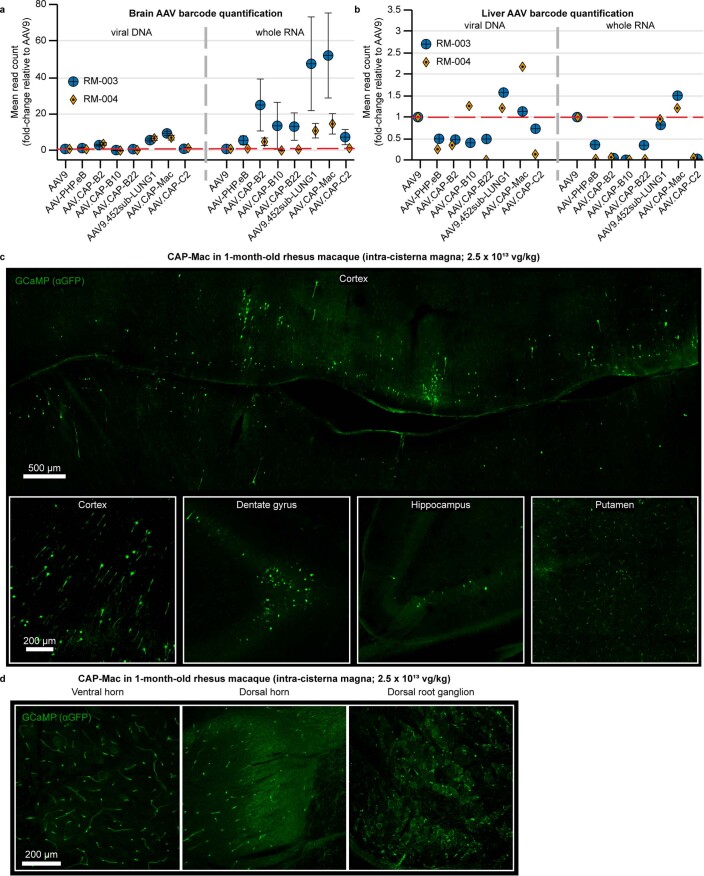
Administering AAV via intra-cisterna magna administration. **a**, **b**, Barcode quantification in viral DNA and whole RNA from brain (**a**) and liver (**b**) of neonate rhesus macaques (n = 2 macaques) treated with a capsid pool via intra-cisterna magna administration. Mean ± s.e.m. shown. **c**, **d**, CAG-GCaMP7s expression in brain (**c**) and spinal cord (**d**) after intra-cisterna magna administration using AAV.CAP-Mac. Source data

Because CAP-Mac outperformed AAV9 and other engineered variants in our pool study, we moved forward with single characterization in two OWP species. In the newborn macaque, we administered a cocktail of three CAP-Mac vectors packaging different fluorescent reporters under the control of a CAG promoter. Fluorescent protein (XFP) expression was observed in multiple coronal slices along the anterior–posterior axis and was robust in all four cortical lobes and in subcortical areas like the dorsal striatum and hippocampus (Fig. 3a). Although expression was particularly strong in several nuclei of the thalamus (for example, lateral and medial nuclei, lateral geniculate nucleus, pulvinar nucleus), expression was not observed in all brain regions (for example, the amygdala). Even with a ubiquitous promoter, we observed expression primarily in NeuN+ neurons and rarely in S100β+ astrocytes (Fig. 3b and Extended Data Fig. 1). We also attempted to deliver CAP-Mac via ICM administration in newborn rhesus macaques, but found that efficiency throughout the brain was noticeably decreased compared with IV administration (Extended Data Fig. 2). Expression was especially low in subcortical structures after ICM administration, as reported previously^27–31^.

**Extended Data Fig. 3:**
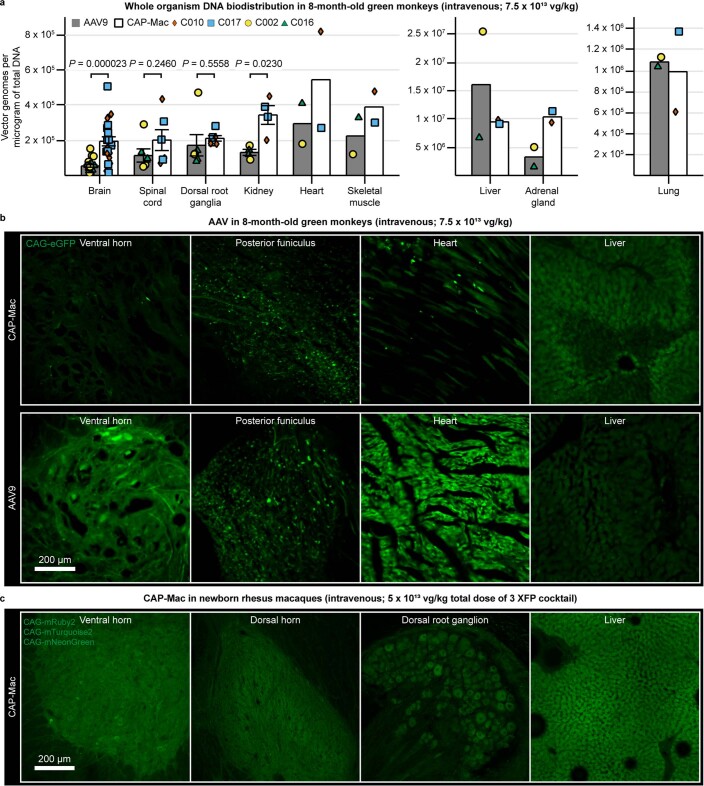
CAG-XFP expression in non-brain tissue of Old World primates treated with AAV. **a**, Vector genomes per microgram of total DNA in green monkeys treated with AAV9 (n = 2 green monkeys) or CAP-Mac (n = 2 green monkeys), expressed as fold-change relative to mean AAV9. Each data point represents measured vector genomes per microgram of total DNA in a section of tissue from each region and monkey. Mean ± s.e.m. shown. Two-tailed Welch’s t-test. **b**, CAG-eGFP expression in the spinal cord, heart, and liver of green monkeys after intravenous expression of either CAP-Mac (top) or AAV9 (bottom). **c**, CAG-XFP expression in the spinal cord, dorsal root ganglia, and liver of newborn rhesus macaque after intravenous administration of CAP-Mac packaging a cocktail of 3 CAG-XFPs. XFPs are pseudocoloured identically. Source data

AAV variants engineered for BBB crossing in mice are known to have strain-dependent behaviour^16,25,38–40^. Therefore, in parallel with the macaque experiments, we characterized CAP-Mac in green monkeys, another OWP species. We administered either AAV9 or CAP-Mac packaging green fluorescent protein (GFP) under the control of CAG (ssCAG-eGFP) to individual monkeys. In the CAP-Mac-dosed monkeys, we saw broad and strong expression in the cortical and various subcortical regions, including the putamen (Fig. 3c), consistent with our observations in macaques (Figs. 2a and 3a,b). Additionally, we saw particularly strong eGFP expression throughout the cerebellum. CAP-Mac eGFP expression was primarily found in neurons and not astrocytes, except in the thalamus where 42% of GFP+ cells were neurons and 51% astrocytes (Fig. 3d). In AAV9-dosed monkeys, AAV9 eGFP expression was primarily biased towards astrocytes in the cortex with low neuronal transduction (Fig. 3e), which is consistent with other reports^27,31,41,42^. In particular, CAP-Mac-recovered transgenes were more abundant throughout the brain compared with AAV9, suggesting overall higher brain penetrance of CAP-Mac (Fig. 3f and Extended Data Fig. 3a). Interestingly, the cerebellum contained the fewest vector genomes (vg) per microgram of DNA in both CAP-Mac-dosed monkeys despite the strong eGFP expression, probably due to the high density of cells and processes within the cerebellum^43,44^. In most non-brain tissue, eGFP biodistribution and expression was comparable between CAP-Mac- and AAV9-treated animals (Extended Data Fig. 3). It should be noted that the cell-type tropism differences we observed between CAP-Mac and AAV9 in the brain may apply to other tissues as well. Given the reported variability in viral infection even in homogeneous cell populations^45–47^, conclusions about capsid penetrance based on viral genomes in whole-tissue extracts may be confounded by these observed cell-type tropism differences.

## Experimental utility of CAP-Mac to study the macaque brain

**Fig. 4:**
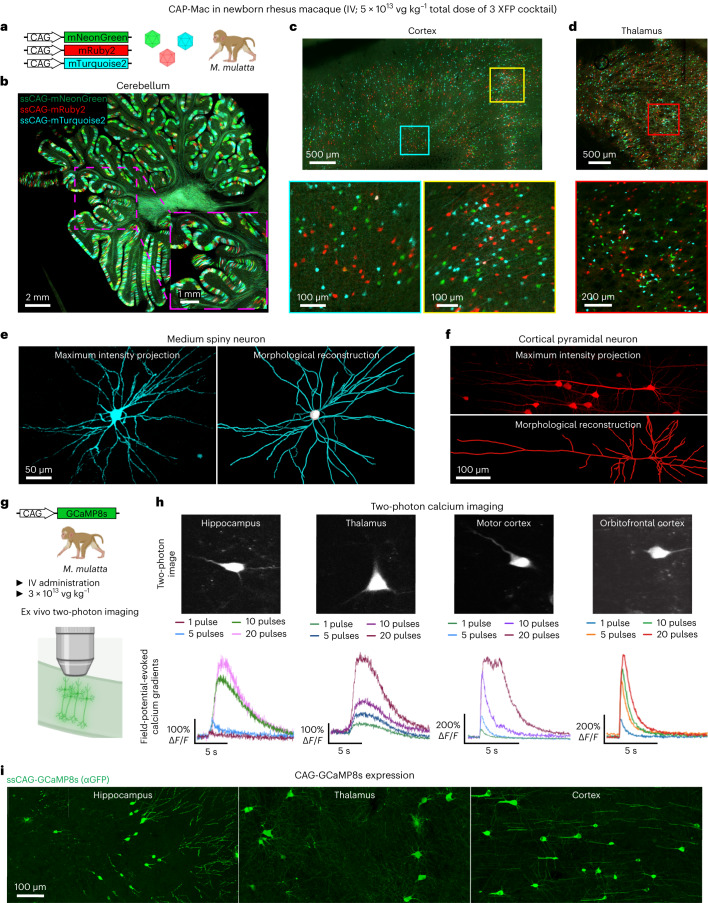
Experimental utility of CAP-Mac for interrogation of the newborn rhesus macaque brain. **a**–**f**, CAP-Mac packaging three fluorescent reporters (**a**) to generate Brainbow-like labelling in rhesus macaque cerebellum (**b**), cortex (**c**) and thalamus (lateral geniculate nucleus) (**d**), enabling morphological reconstruction of neurons (**e** and **f**). **g**–**i**, Non-invasive delivery of ssCAG-GCaMP8s using CAP-Mac (**g**) for ex vivo two-photon imaging (**h**) and brain-wide GCaMP expression (**i**).

The BRAIN initiative emphasizes the importance of developing novel tools for genetic modulation in NHPs to inform further understanding of the human brain^48^. Accordingly, we leveraged CAP-Mac’s neuronal tropism in newborn macaques to deliver genetically encoded reporters to interrogate the NHP brain. First, we used CAP-Mac as a non-invasive method to define neuronal morphology. Having administered a cocktail of three CAP-Mac vectors packaging different XFPs, we attempted Brainbow-like labelling^13,34,35^ in an OWP (Fig. 4a–d). We observed widespread expression of all three XFPs throughout the brain, including the cerebellum, cortex and lateral geniculate nucleus of the thalamus. In the cerebellum and thalamus, we observed a high density of transduced cells, and the highest proportion of co-localization of two or three XFPs. Given this robust expression, we were able to reconstruct the morphology of medium spiny neurons and cortical pyramidal cells (Fig. 4e,f).

**Extended Data Fig. 4:**
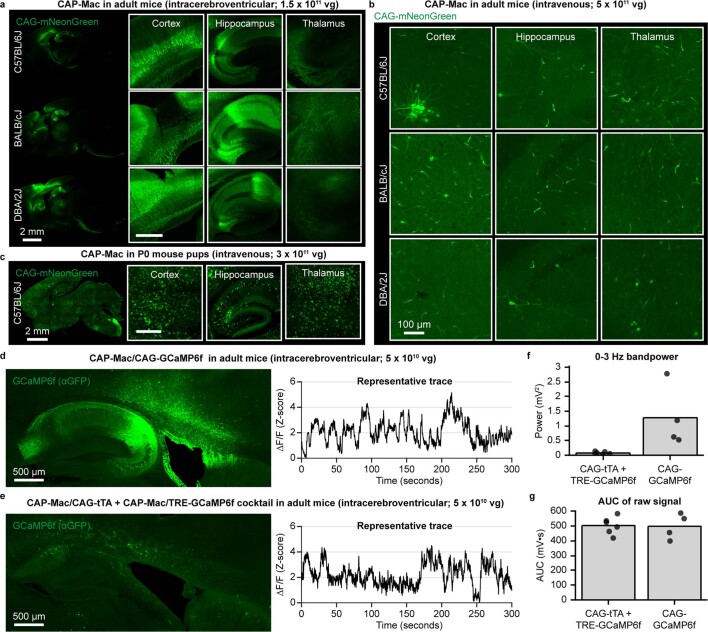
Tropism in mice and utilizing mice as a model organism for cargo validation. **a**, CAP-Mac after intracerebroventricular (ICV) administration in adult mice primarily transduces neurons, mimicking the CAP-Mac tropism in infant Old World primates after intravenous (IV) administration. **b**, CAP-Mac after IV administration in C57BL/6 J, BALB/cJ, and DBA/2 J adult mice primarily transduces vasculature, with no apparent differences between the three mouse strains. **c**, CAP-Mac in P0 C57BL/6 J pups after intravenous administration transduces various cell-types, including neurons, astrocytes, and vasculature. **d-g**, Given the neuronal tropism of CAP-Mac via ICV administration, we validated GCaMP cargo in mice prior to non-human primate experiments, testing either one-component or two-component systems. **d**, **e**, GCaMP protein expression and representative ΔF/F traces in mice after delivering CAG-GCaMP6f (n = 2 mice) (**d**) or a CAG-tTA/TRE-GCaMP6f cocktail (n = 3 mice) using CAP-Mac (**e**). **f**, **g**, To determine cargo to move forward with, we found that 0-3 Hz bandpower (two-tailed Welch’s t-test, P = 0.105) (**f**), but not area under the curve (AUC; two-tailed Welch’s t-test, P = 0.626) (**g**), was indicative of cargo performance.

In a second set of experiments, we used CAP-Mac to express functional GCaMP throughout the infant rhesus macaque brain (Fig. 4g). Given the experimental complexity and limited availability of NHPs, we performed initial cargo screening in mice, testing GCaMP configurations in single- and dual-vector formats (Extended Data Fig. 4). Based on our results, we found that the neuronal bias of CAP-Mac extended to mice when delivered to the brain via intracerebroventricular (ICV), but not IV, administration and opted to use a one-component vector using the CAG promoter in NHPs.

**Extended Data Fig. 5:**
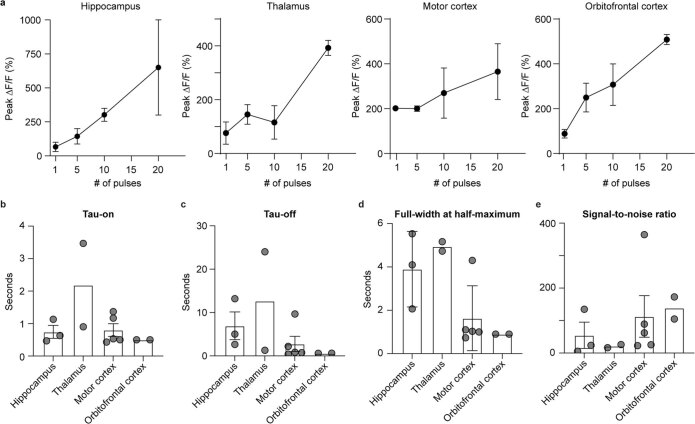
Group-level analyses of two-photon calcium imaging in rhesus macaque slice. **a**, Mean peak ΔF/F_0_ evoked by cells from hippocampus, thalamus, motor cortex, and orbitofrontal cortex after applying different numbers of pulses. **b**, Mean rise time of GCaMP8s responses in the four brain regions. Rise time is defined as time taken for the response to rise from 10% to 90% of the peak of the amplitude. **c**, Mean decay time constant of GCaMP8s responses in the four brain regions. Decay time constant was obtained by fitting sums of exponentials to the decay phase of the traces. **d**, Mean full width at half maximum (FWHM) of GCaMP8s responses in the four brain regions. **e**, Mean signal-to-noise ratio (SNR) of GCaMP8s responses in the four brain regions. SNR is defined as the peak amplitude divided by the standard deviation of the fluorescence signal before the electrical stimulation. Hippocampus: n = 3 cells. Thalamus: n = 2 cells. Motor cortex: n = 5 cells. Orbitofrontal cortex: n = 2 cells. Data is plotted as mean ± s.e.m.

We intravenously delivered ssCAG-GCaMP8s to newborn macaques (3 × 10^13^ vg kg^−^^1^ via the saphenous vein) and, after 4–6 weeks of expression, isolated tissue for ex vivo two-photon imaging. In the hippocampus, thalamus and cortex, we successfully recorded the field-potential-evoked calcium gradients of GCaMP-expressing cells (Fig. 4h). Cells were responsive to restimulation throughout the experiment and, importantly, the mean peak Δ*F*/*F* of the GCaMP signal increased with an increasing number of field potential pulses, with differing cellular calcium dynamics across the four sampled brain regions (Extended Data Fig. 5). Consistent with our previous profiling, we saw GCaMP expression primarily in cell types with neuronal morphology throughout the brain (Fig. 4i).

## CAP-Mac strongly transduces cultured human neurons

**Fig. 5:**
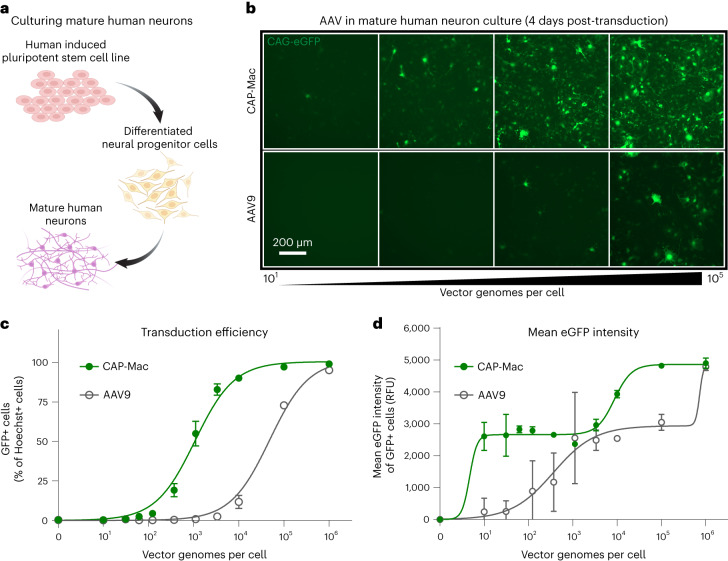
CAP-Mac is more potent in human cultured neurons compared with AAV9. **a**, Differentiation process starting with an iPSC line that was differentiated into neural progenitor cells, which were further differentiated into mature neurons. **b**, Representative images of cultured human neurons after 4 days of incubation with either CAP-Mac (top) or AAV9 (bottom) packaging ssCAG-eGFP across four doses of AAV, ranging from 10^1^ to 10^5^ vg per cell. **c**,**d**, Dose response curves of AAV9 (*n* = 3 biologically independent replicates) and CAP-Mac (*n* = 3 biologically independent replicates) in mature human neuron cultures measuring the transduction efficiency (**c**) and mean eGFP intensity (**d**). Mean values ± s.e.m. are shown. Source data

Given the efficacy of CAP-Mac in penetrating the brain and its neuronal bias in infant OWPs, we wanted to test whether CAP-Mac offered any improvement over its parent capsid, AAV9, in transducing human neurons. We differentiated cultured human-derived induced pluripotent stem cells (iPSCs) into mature neurons (Fig. 5a) and incubated them with CAP-Mac or AAV9 packaging ssCAG-eGFP at doses ranging from 0 to 10^6^ vg per cell. We found that the eGFP expression was noticeably higher in CAP-Mac-administered cultures than AAV9-administered cultures (Fig. 5b). AAV9 transduction achieved an efficiency of EC_50_ = 10^4.68^ vg per cell, whereas CAP-Mac achieved EC_50_ = 10^3.03^ vg per cell (Fig. 5c), a 45-fold increase in potency (*P* = 0.0023 using two-tailed Welch’s *t*-test). The average per-cell eGFP expression measured across transduced cells fits a biphasic step function, with CAP-Mac reaching the first plateau at a dose roughly two orders of magnitude lower than AAV9 (Fig. 5d). Overall, the increased potency of CAP-Mac in transducing mature human neurons in vitro is consistent with the neuronal tropism that we observed in infant OWPs, suggesting a similar mechanism of neuronal transduction across species.

## An improved systemic vector over AAV9 in adult NHP tissue

**Fig. 6:**
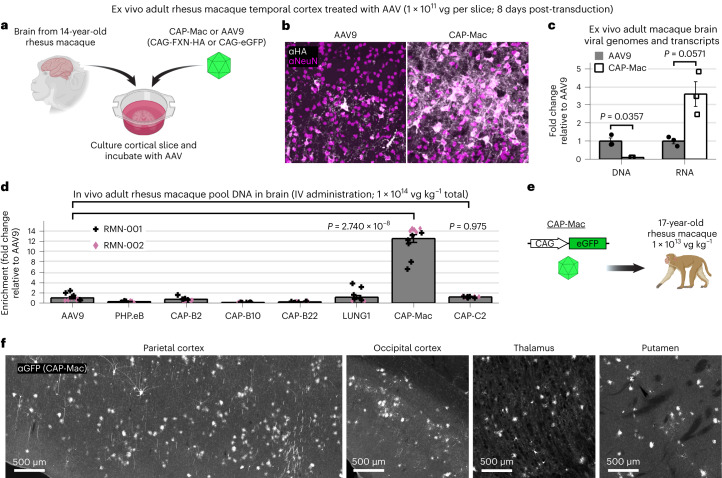
Characterization in adult rhesus macaque. **a**, AAV in ex vivo cortical slice taken from a 14-year-old rhesus macaque. **b**, CAP-Mac is more efficient at transducing neurons in the grey matter of the cortex. **c**, Quantification demonstrates that CAP-Mac-delivered transgene produces more RNA but not DNA compared with AAV9-delivered transgene; *n* = 3 brain slices; two-tailed Welch’s *t*-test. **d**–**f**, AAV in adult rhesus macaques in vivo. **d**, Recovered DNA from adult macaque administered with an eight-capsid pool (7.5 × 10^13^ vg kg^−^^1^). Here *n* = 12 brain samples from two adult macaques. One-way analysis of variance using Tamhane’s T2 correction was tested against AAV9 enrichment. **e**, We intravenously injected 1 × 10^13^ vg of CAP-Mac packaging CAG-eGFP into a 17-year-old rhesus macaque via the saphenous vein to assess CAP-Mac protein expression in the adult macaque. **f**, CAP-Mac-mediated eGFP expression visualized after amplification with GFP antibody. Mean values ± s.e.m. are shown. Source data

Infant NHPs offer several logistical advantages for AAV characterization: they are more likely to be seronegative for neutralizing AAV antibodies, and their smaller body mass requires fewer vectors to be produced. However, although the mammalian BBB is fully formed by birth—including intact tight junctions that give rise to the BBB’s unique functionality to limit passive molecular transport into the brain—dynamic molecular and cellular processes during development may make the BBB more permissive in infants^49–52^. Therefore, we characterized CAP-Mac’s tropism in adult macaque tissue. To de-risk our approach, we chose to first test CAP-Mac in adult rhesus macaque slices ex vivo (Fig. 6a). In the grey matter of cultured cortical slices, cargo delivered by CAP-Mac, but not AAV9, co-localized with NeuN+ cells, consistent with our previous results (Fig. 6b). Unexpectedly, only 9% as many CAP-Mac viral genomes were recovered as AAV9 genomes, but 3.6-fold more viral transcripts were recovered from CAP-Mac-treated slices than from AAV9-treated slices (Fig. 6c).

Although informative, ex vivo characterization does not assess BBB penetration, so next we tested CAP-Mac in adult macaques in vivo. We injected two adult rhesus macaques with the same AAV pool used in infants and found 13-fold more CAP-Mac-delivered genomes in the brain than AAV9 (Fig. 6d). Again, all the variants originally selected in mice were less efficient than AAV9, but CAP-C2 was 1.2-fold more efficient than AAV9. To further assess protein expression, we injected CAP-Mac packaging ssCAG-eGFP into a 17-year-old adult rhesus macaque (Fig. 6e). At the protein level, we observed CAP-Mac-delivered eGFP expression (visualized by eGFP antibody amplification) in parts of the cortex and thalamus, but not in other regions of the brain (Fig. 6f).

**Extended Data Fig. 6:**
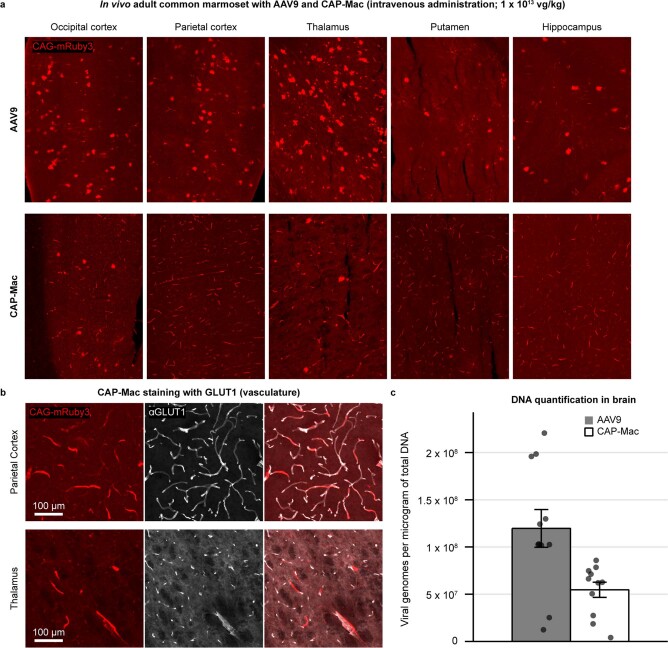
CAP-Mac tropism in adult common marmoset compared to AAV9. **a**, AAV9 and CAP-Mac tropism in two adult marmosets in vivo (3.8- and 5.8-years-old). **b**, CAP-Mac is biased primarily towards GLUT1+ cells (vasculature), consistent with our results in adult mice. **c**, Recovered viral genomes in adult marmoset brain (n = 2 marmosets). Mean ± s.e.m. shown. Two-tailed Welch’s t-test, P = 0.00981. Source data

Finally, since CAP-Mac was originally identified using in vivo selections in the adult common marmoset, we also wanted to characterize the vector in the selection species. As in the adult macaque experiment, we injected CAP-Mac and AAV9 into adult marmosets (3.8 and 5.8 years old). To our surprise, we found that the tropism of CAP-Mac in adult marmoset was primarily biased towards the GLUT1+ vasculature (Extended Data Fig. 6).

## Conclusions

Here we describe AAV.CAP-Mac, an engineered AAV9 variant with increased efficiency for brain-wide transgene expression in multiple NHP species.

By characterizing CAP-Mac in various experimental contexts, we found that tropism varies depending on the species, developmental stage and route of administration (Supplementary Table 1). This is not surprising given the heterogeneity of the BBB across species and populations^53–55^, a challenge noted in other AAV engineering efforts^56–59^. Performing such a comprehensive characterization of CAP-Mac in multiple contexts was beneficial for two reasons. First, we discovered that CAP-Mac was primarily biased towards the brain endothelium in the adult marmoset, which is inaccessible with the current toolbox of AAV vectors. Second, by testing multiple routes of administration, we found that CAP-Mac tropism is shifted towards neurons after ICV administration, mirroring the tropism in newborn macaques and giving us a method to assess expression and functional activity of GCaMP constructs before applying them to NHPs (Extended Data Fig. 4). In lieu of a cross-species capsid with conserved tropism and efficiency in rodents and NHPs, this approach was a valuable method to validate capsid–cargo combinations in mice before use in NHPs.

In vivo AAV capsid selections have been primarily conducted in mice, in part due to the availability of Cre-transgenic mouse lines to increase selective pressure, which can yield neurotropic capsids in as few as two rounds of selection^12,15,16^. However, these engineered variants have mostly failed to translate to NHPs^24,25^. Although mice last shared a common ancestor with humans approximately 80–90 million years ago, marmosets and macaques shared their last ancestors with humans 35–40 and 25–30 million years ago, respectively^23,60^. Given this evolutionary distance, it is not surprising that variants selected in mice have failed to translate to OWPs, and vice versa. As such, the success of the variants selected in marmosets described here (namely, CAP-Mac and CAP-C2) compared with the variants selected in rodents highlights the importance of considering the evolutionary relatedness between the selection and target species.

**Extended Data Fig. 7:**
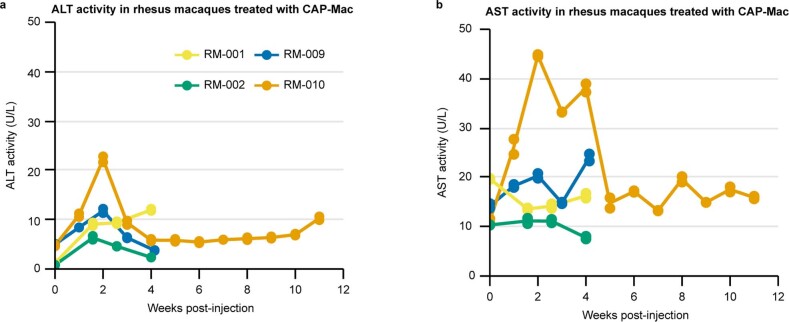
Liver function tests in newborn rhesus macaques treated with AAV. **a**, **b**, Liver function tests show no abnormal signs of adverse liver functionality, as measured by alanine transaminase (ALT; **a**) and aspartate transaminase (AST; **b**) activity. Source data

The overarching goal of this study was to define and disseminate a suite of genetic tools to study the NHP brain, especially in OWPs. To that end, we describe two functional cargos for studying the OWP brain: (1) a cocktail of three fluorescent reporters for Brainbow-like^34,35^ labelling and (2) GCaMP8s for the optical interrogation of ex vivo neuronal activity. We note here that in the Brainbow experiment, the co-localization of multiple XFPs was rare, suggesting that co-infection was uncommon after systemic administration. This may have broad implications for the clinical applicability of dual-vector approaches that require the co-infection of heterodimeric AAVs to express large protein products^61–64^. Encouragingly, our GCaMP recordings demonstrate that cells expressing CAP-Mac-delivered molecular sensors are physiologically active and healthy in ex vivo macaque slices. Together, these experiments demonstrate that CAP-Mac now enables non-invasive, systemic delivery of genetically encoded sensors to the macaque brain, a transformative technique previously limited to rodents. In particular, none of the macaques dosed in this study experienced adverse events or abnormal liver function, and an assessment by an independent pathologist confirmed that the vectors were safely administered (Extended Data Fig. 7 and Supplementary Table 2). Moving forward, we expect CAP-Mac-mediated gene transfer to help illuminate circuit connectivity and neuronal function in the macaque brain^65,66^ and, more generally, assist major efforts such as the BRAIN initiative^48^ to understand the inner workings of the primate CNS.

In addition to CAP-Mac’s utility as a tool to study the primate brain, it is also a compelling potential gene therapy vehicle in humans. It provides an unprecedented opportunity to deepen our understanding of pharmacodynamics in OWP models^67–69^ and its broad and uniform distribution throughout the CNS opens access to subcortical and midbrain regions for neuroscientists, currently difficult in NHPs^27–31^. Additionally, CAP-Mac’s enhanced transduction of cultured human neurons supports its potential as a clinical gene-delivery vehicle. Overall, the success of the capsid engineering approach we describe here offers a roadmap for developing the next class of translational gene therapies with improved safety and efficacy profiles.

## Methods

All the experiments and procedures were approved by local regulatory boards and committees and were required to comply with study protocols. All the mouse procedures were performed at Caltech, approved by the Caltech Institutional Animal Care and Use Committee (IACUC; protocol 1738). Marmoset (protocol TGC-03) and adult macaque (protocol LN-14) procedures took place at the NIH and were approved by the NIH IACUC. Marmoset procedures were also completed at University of California San Diego (UCSD) (protocol S09147) and were in compliance with and approved by the UCSD IACUC. Infant macaque procedures took place at the California National Primate Research Center at University of California Davis, and were approved by their local IACUC (protocol 22525). Green monkey procedures took place at Virscio and were approved by their local IACUC.

### AAV DNA library generation

The details of this procedure can be found on protocols.io (10.17504/protocols.io.5jyl8jy89g2w/v1). We initially generated diversity at the DNA level, which we then used to produce transfection material to produce the AAV capsid library, as described previously in detail^16^. For the first-round library, we introduced this genetic diversity using primers containing degenerate nucleotides inserted between amino acids 588 and 589 (refs. ^12,13,16^) (VP1 numbering; Supplementary Fig. 1a). We used a reverse primer containing 7 degenerate nucleotides ([NNK] × 7) to randomly generate polymerase chain reaction (PCR) fragments containing unique 7mer sequences inserted into the *cap* gene. For the second-round DNA library, we used a synthetic oligo pool (Twist Bioscience) as a reverse primer, encoding only variants selected for further screening (total, 66,628 DNA oligos; 33,314 variants recovered after first-round selections plus a codon-modified replicate of each). All the reverse primers contained a 20 bp 5′ overhang complementary to the *cap* sequence near the AgeI restriction enzyme sequence and were paired with a forward primer containing a 20 bp 5′ overhang near the XbaI restriction enzyme sequence. We then inserted the PCR fragments containing the diversified region into the rAAV-ΔCAP-in-cis-Lox plasmid via Gibson assembly to generate the resulting AAV DNA library, namely, rAAV-CAP-in-cis-Lox, using NEBuilder HiFi DNA Assembly Master Mix (New England Biolabs, E2621).

### AAV capsid library production

The details of this procedure can be found on protocols.io (10.17504/protocols.io.5jyl8jyz9g2w/v1). We generated AAV capsid libraries according to previously published protocols^16,70^. Briefly, we transfected HEK293T cells (ATCC, cat # CRL-3216; RRID: CVCL_0063) in 150 mm tissue culture plates using transfection-grade linear polyethylenimine (PEI; Polysciences). In each plate, we transfected four plasmids: (1) the assembled rAAV-Cap-in-cis-Lox AAV DNA library, which is flanked by inverted terminal repeats required for AAV encapsidation; (2) AAV2/9 REP-AAP-ΔCAP, which encodes the REP and AAP supplemental proteins required for AAV production with the C terminus of the *cap* gene excised to prevent recombination with the AAV DNA library and subsequent production of replication-competent AAV; (3) pHelper, which encodes the necessary adenoviral proteins required for AAV production; and (4) pUC18 (Addgene ID: 50004; RRID: Addgene_50004), which contains no mammalian expression vector but is used as filler DNA to achieve the appropriate nitrogen-to-phosphate ratio for optimal PEI transfection. During preparation of the PEI–DNA mixture, we added 10 ng of our AAV DNA library (rAAV-Cap-in-cis-Lox) for every 150 mm dish and combined AAV2/9 REP-AAP-ΔCAP, pUC18 and pHelper in a 1:1:2 ratio (40 µg of total DNA per 150 mm dish). At 60 h post-transfection, we purified the AAV capsid library from both cell pellet and media using polyethylene glycol precipitation and iodixanol gradient ultracentrifugation. Using quantitative PCR, we then determined the titre of the AAV capsid libraries by amplifying DNaseI-resistant viral genomes relative to a linearized genome standard according to established protocols^70^.

### Marmoset experiments

#### Capsid library selections

The details of this procedure can be found on protocols.io (10.17504/protocols.io.bp2l695zklqe/v2). All the marmoset (*C. jacchus*) procedures were performed at the National Institute of Mental Health (NIMH) and approved by the local IACUC. Marmosets were born and raised in NIMH colonies and housed in family groups under standard conditions of 27 °C and 50% humidity. They were fed ad libitum and received enrichment as part of the primate enrichment program for NHPs at the NIH. For all the marmosets used in this study, there were no detectible neutralizing antibodies at a 1:5 serum dilution before IV infusions (assayed by the Penn Vector Core, University of Pennsylvania). They were then individually housed for several days and acclimated to a new room before injections. Four adult males were used for the library screening, two each for the first- and second-round libraries. The day before infusion, the animals’ food was removed. Animals were anaesthetized with isoflurane in oxygen, the skin over the femoral vein was shaved and sanitized with an isopropanol scrub and 2 × 10^12^ vg of the AAV capsid library was infused over several minutes. Anaesthesia was withdrawn and the animals were monitored until they became active, on which they were returned to their cages. Activity and behaviour were closely monitored over the next 3 days, with daily observations thereafter.

At 4 weeks post-injection, marmosets were euthanized (Euthanasia, VetOne) and perfused with 1× phosphate-buffered saline (PBS). After the first-round library, the brain was cut into four coronal blocks, flash frozen in 2-methylbutane (Sigma-Aldrich, M32631), chilled with dry ice and stored at −80 °C for long-term storage. After the second-round library, the brain was cut into six coronal blocks and, along with sections of the spinal cord and liver, was flash frozen and stored at −80 °C for long-term storage.

#### Individual characterization of AAVs in marmosets

The details of the procedures in this section can be found on protocols.io (10.17504/protocols.io.5qpvormddv4o/v1 and 10.17504/protocols.io.j8nlkwxxwl5r/v1). Two adult common marmosets (*C. jacchus*) were used for this experiment: Conan (male, 2.8 years old, 0.386 kg) and Sandy (female, 5.8 years old, 0.468 kg) (Supplementary Table 3 provides more details). They were housed under standard conditions of 27 °C and 50% humidity, with ad libitum access to food and water. All the animals were group housed, and the experiments were performed in the Cortical Systems and Behavior Laboratory at UCSD. All the experiments were approved by the UCSD IACUC. The day before infusion, the animals’ food was removed.

Animals were anaesthetized with ketamine (Ketaset, Zoetis 043-304, 20 mg kg^−^^1^), the skin over the saphenous vein was shaved and sanitized with an isopropanol scrub and 2 × 10^13^ vg kg^−^^1^ of AAV was infused over 5 min. The animals were monitored until they became active, on which they were returned to their cages. Activity and behaviour were closely monitored over the next 3 days, with daily observations thereafter. Blood samples were taken on days 1, 7, 14, 21 and 31 to measure the viral concentration in plasma.

At 31 days post-injection, the marmosets were anaesthetized with ketamine as described earlier and then euthanized (Euthasol, Virbac 200-071, 1 ml kg^−^^1^) and perfused with 1× PBS. Brains and organs were cut in half, and one half was flash frozen in 2-methylbutane (Sigma-Aldrich, M32631), chilled with dry ice and stored at −80 °C. The other half was fixed in 4% paraformaldehyde (PFA) (Thermo Scientific, J19943-K2) overnight and then stored at 4 °C in PBS azide (Sigma-Aldrich, S2002-100G, 0.025%). The samples were then shipped to the California Institute of Technology (Caltech) for analysis. For GLUT1 staining, we incubated slices with rabbit anti-GLUT1 (1:200; Millipore-Sigma, cat # 07-1401; RRID: AB_1587074), performed three to five washes with PBS, incubated with donkey anti-rabbit IgG (1:200; Jackson ImmunoResearch Labs, cat # 711-605-152; RRID: AB_2492288) and washed three to five times before mounting. We diluted all antibodies and performed all incubations using PBS supplemented with 0.1% Triton X-100 (Sigma-Aldrich, T8787) and 10% normal donkey serum (Jackson ImmunoResearch Labs, cat # 017-000-121; RRID: AB_2337258) overnight at room temperature with shaking.

### Viral library DNA extraction and NGS sample preparation

The details of this procedure can be found on protocols.io (10.17504/protocols.io.bp2l695zklqe/v2). We previously reported that viral library DNA and endogenous host RNA can be isolated using TRIzol by precipitating nucleic acid from the aqueous phase^12,16^. Therefore, to extract viral library DNA from marmoset tissue, we homogenized 100 mg of spinal cord, liver and each coronal block of the brain in TRIzol (Life Technologies, 15596) using a BeadBug (Benchmark Scientific, D1036) and isolated nucleic acids from the aqueous phase according to the manufacturer’s recommended protocol. We treated the reconstituted precipitate with RNase (Invitrogen, AM2288) and digested with SmaI to improve downstream viral DNA recovery via PCR. After digestion, we purified with a Zymo DNA Clean and Concentrator kit (D4033) according to the manufacturer’s recommended protocol and stored the purified viral DNA at −20 °C.

To append Illumina adaptors flanking the diversified region, we first PCR amplified the region containing our 7mer insertion using 50% of the total extracted viral DNA as a template (25 cycles). After Zymo DNA purification, we diluted the samples at 1:100 and further amplified around the variable region with ten cycles of PCR, appending binding regions for the next PCR reaction. Finally, we appended Illumina flow cell adaptors and unique indices using NEBNext Dual Index Primers (New England Biolabs, E7600) via ten more cycles of PCR. We then gel purified the final PCR products using a 2% low-melting-point agarose gel (Thermo Fisher Scientific, 16520050) and recovered the 210 bp band.

For the second-round library only, we also isolated the encapsidated AAV library ssDNA for NGS to calculate the library enrichment scores, a quantitative metric that we used to normalize for differences in titre of the various variants in our library (see ref. ^16^ and the ‘NGS read alignment, analysis and generation of network graphs’ section). To isolate the encapsidated viral genomes, we treated the AAV capsid library with DNaseI and digested capsids using proteinase K. We then purified the ssDNA using phenol–chloroform, amplified viral transgenes by two PCR amplification steps to add adaptors and indices for Illumina NGS and purified using gel electrophoresis. This viral library DNA, along with the viral DNA extracted from the tissue, was sent for deep sequencing using an Illumina HiSeq 2500 system (Millard and Muriel Jacobs Genetics and Genomics Laboratory, Caltech).

### NGS read alignment, analysis and generation of network graphs

Raw FASTQ files from NGS runs were processed with custom-built scripts (https://github.com/GradinaruLab/protfarm and https://github.com/GradinaruLab/mCREATE)^16^. For the first-round library, the pipeline to process these datasets involved filtering to remove low-quality reads, utilizing a quality score for each sequence, and eliminating bias from PCR-induced mutations or high GC-content. The filtered dataset was then aligned by a perfect string match algorithm and trimmed to improve the alignment quality. We then displayed the absolute read counts for each variant during the sequencing run within each tissue, and all the 33,314 variants that were found in the brain were chosen for the second-round selections.

After the second-round selections, we performed the same analysis to display the variant absolute read count of the injected virus library and of each variant within each tissue. Additionally, we calculated the library enrichment^16^ for each variant within each tissue:

1 $${\overline{{\rm{RC}}}}_{x,{\rm{injected}}\,{\rm{library}}}=\,\frac{{{\rm{RC}}}_{x,{\rm{injected}}\,{\rm{library}}}}{\mathop{\sum }\nolimits_{i=1}^{{N}_{{\rm{injected}}\,{\rm{library}}}}{{\rm{RC}}}_{i,{\rm{injected}\,{library}}}},$$

2 $${\overline{{\rm{RC}}}}_{x,{\rm{tissue}}}=\,\frac{{{\rm{RC}}}_{x,{\rm{virus}}}}{\mathop{\sum }\nolimits_{i=1}^{{N}_{{\rm{tissue}}}}{{\rm{RC}}}_{i,{\rm{tissue}}}},$$

3 $${\rm{Library}\,{enrichment}}=\,{\log }_{10}\left(\frac{{\overline{{\rm{RC}}}}_{x,\rm{{injected}\,{library}}}}{{\overline{{\rm{RC}}}}_{x,{\rm{tissue}}}}\right),$$

such that for a given sample *y* (for example, the injected virus library or a tissue sample), RC_*x*,*y*_ is the absolute read count of variant *x*, *N*_*y*_ is the total number of variants recovered and ${\overline{{\rm{RC}}}}_{x,{y}}$ is the normalized read count.

To construct the CAP-Mac sequence clustering graph, we filtered the second-round NGS data based on the following criteria: (1) ≥100 read count in the injected library sample (24,186/33,314 variants), (2) ≥0.7 library enrichment score in more than two brain samples (415 variants) and (3) at least two more brain samples with ≥0.7 library enrichment than brain samples with less than −0.7 library enrichment (323 variants). To construct the CAP-C2 sequence graph, we filtered the second-round NGS data based on the following criteria: (1) ≥100 read count in the injected library sample and (2) both codon replicates present in at least two brain samples with ≥0.7 library enrichment (95 variants). These variants were then independently processed to determine pair-wise reverse Hamming distances (https://github.com/GradinaruLab/mCREATE) and clustered using Cytoscape (v. 3.9.0; RRID: SCR_003032) as described previously in detail^16^. Networks presented show capsid variants (nodes) connected by edges if the pair-wise reverse Hamming distance is ≥3.

### Cloning individual AAV capsid variants

The details of this procedure can be found on protocols.io (10.17504/protocols.io.n2bvj87ebgk5/v1). For single-variant characterization, we cloned new variant plasmids by digesting a modified version of the pUCmini-iCAP-PHP.eB (Addgene ID: 103005; RRID: Addgene_103005) backbone using MscI and AgeI. We designed a 100 bp primer that contained the desired 21 bp insertion for each capsid variant and the regions complementary to the AAV9 template with ~20 bp overlapping the digested backbone. We then assembled the variant plasmid using NEBuilder HiFi DNA Assembly Master Mix, combining 5 μl of 200 nM primer with 30 ng of digested backbone in the reaction mixture. The capsid plasmid used to produce AAV.CAP-Mac is available on Addgene (Addgene ID: 200658; RRID: Addgene_200658).

### Individual AAV production and purification

The details of this procedure can be found on protocols.io (10.17504/protocols.io.14egn2dqzg5d/v1). To produce variants for pool testing, we followed our previously published protocol^70^ using 150 mm tissue culture dishes. For individual AAV.CAP-Mac and AAV9 characterization in vivo and in vitro, we adopted our published protocol to utilize ten-layer CellSTACKs (Corning, 3320) to efficiently produce viruses at a high titre to dose rhesus macaques and green monkeys. Specifically, we passaged twenty 150 mm dishes at approximately 70% confluency into a ten-layer CellSTACK 24 h before transfection. On the day of the transfection, we prepared the PEI–DNA transfection mixture for forty 150 mm dishes and combined the transfection mixture with media and performed a complete media change for the CellSTACK. We collected and changed the media at 72 h post-transfection similar to production in 150 mm dishes. At 120 h post-transfection, we added ethylenediaminetetraacetic acid (Invitrogen, 15575020) to a final concentration of 10 mM and incubated at 37 °C for 20 min, occasionally swirling and tapping the sides of the CellSTACK to detach the cells. We then removed the media and cell mixture and proceeded with the AAV purification protocol^70^. Of note, during the buffer exchange step after ultracentifugation, we used centrifugal protein concentrators with polyethersulfone membranes (Thermo Scientific, 88533) instead of Amicon filtration devices and used Dulbecco’s PBS supplemented with 0.001% Pluronic F-68 (Gibco, 24040032).

### Rodent experiments

All the rodent procedures were performed at Caltech and were approved by the local IACUC. We purchased C57BL/6J (strain #: 000664; RRID: IMSR_JAX:000664), BALB/cJ (strain #: 000651; RRID: IMSR_JAX:000651) and DBA/2J (strain #: 000671; RRID: IMSR_JAX:000671) mice (all males, 6–8 weeks old) from The Jackson Laboratory. For IV administration in mice, we delivered 5 × 10^11^ vg of virus through the retro-orbital sinus^70,71^ using a 31 gauge insulin syringe (BD, 328438). See protocols.io for more details on retro-orbital injections of AAV in mice (10.17504/protocols.io.3byl4joy8lo5/v1). For intracerebroventricular (ICV) administration in mice, we injected 5.0 × 10^10^ or 1.5 × 10^11^ vg into the lateral ventricle. Briefly, we anaesthetized mice using isoflurane (5% for induction, 1–3% for maintenance) with 95% O_2_/5% CO_2_ (1 l min^−^^1^) and the mice were head fixed in a stereotaxic frame. After shaving the head and sterilizing the area with chlorohexidine, we subcutaneously administered 0.05 ml of 2.5 mg ml^−^^1^ bupivacaine, and a midline incision was made and the skull was cleaned of blood and connective tissue. After levelling the head, burr holes were bilaterally drilled above the lateral ventricles (0.60 mm posterior to bregma and 1.15 mm from the midline). Viral vectors were aspirated into 10 µl NanoFil syringes (World Precision Instruments) using a 33 gauge microinjection needle, and the needle was slowly lowered into the lateral ventricle (1.6 mm from the pial surface). The needle was allowed to sit in place for approximately 5 min and 3–5 µl of viral vector was injected using a microsyringe pump (World Precision Instruments, UMP3) and pump controller (World Precision Instruments, Mircro3) at a rate of 300 nl min^−^^1^. All the mice intraoperatively received 1 mg kg^−^^1^ of buprenorphine SR and 5 mg kg^−^^1^ of ketoprofen subcutaneously and 30 mg kg^−^^1^ of ibuprofen and 60 mg kg^−^^1^ of trimethoprim/sulfamethoxazole for 5 days post-surgery. See protocols.io for more details on ICV injections of AAV in mice (10.17504/protocols.io.5qpvorm4dv4o/v1). After 3 weeks of expression, all the mice were perfused with PBS and fixed in 4% PFA. All the organs were extracted, incubated in 4.00% PFA overnight, transferred into PBS supplemented with 0.01% sodium azide and stored at 4 °C for long-term storage. We sliced the brain into 100 μm sections with a vibratome (Leica Biosystems, VT1200S), mounted in Prolong Diamond Antifade (Invitrogen, P36970) and imaged using a confocal microscope (Zeiss, LSM 880) using ZEN (Black edition). See protocols.io for more details on tissue handling (10.17504/protocols.io.5qpvormddv4o/v1 and 10.17504/protocols.io.j8nlkwxxwl5r/v1).

### Rhesus macaque experiments

The details of the procedures in this section can be found on protocols.io (10.17504/protocols.io.5qpvormddv4o/v1). Neonate macaques (0.45–1.40 kg) were weaned at birth. Within the first month, macaques were infused with AAV vectors either intravenously or intrathecally. All adult macaques (8–17 years old; 4.65–11 kg) included in this study were infused with AAV via IV administration only. For IV injections, the animals were anaesthetized with ketamine (0.10 ml) and the skin over the saphenous vein was shaved and sanitized. AAV (between 2 × 10^13^ and 1 × 10^14^ vg kg^−^^1^) was slowly infused into the saphenous vein over ~1 min in <0.75 ml of PBS. For ICM injections, the animals were intramuscularly administered a sedative and the area of the skin at the neck was shaved and aseptically prepared. A needle was advanced into the cisterna magna to remove a small amount of CSF proportional to the amount of fluid injected. Then, a sterile syringe containing the sterile preparation of the AAV (1.5 × 10^12^ or 2.5 × 10^13^ vg kg^−^^1^) proportional to the amount of fluid collected was aseptically attached and slowly injected. All the animals were monitored during recovery from sedation throughout the day and then daily for any adverse findings. All the monkeys were individually housed within sight and sound of conspecifics. Tissue was collected 4–11 weeks after injection. The animals were deeply anaesthetized and received sodium pentobarbital in accordance with guidelines for humane euthanasia of animals at the California National Primate Research Center. All the material injected into rhesus macaques was free of endotoxins (<0.1 EU ml^−^^1^), and protein purity was confirmed by sodium dodecyl sulphate–polyacrylamide gel electrophoresis. Supplementary Tables 4 and 5 list the route of administration, AAV variants, viral dose, genetic cargo and duration of expression for each experiment.

#### Pool testing in rhesus macaques

The details of the procedures in this section can be found on protocols.io (10.17504/protocols.io.5qpvormddv4o/v1, 10.17504/protocols.io.3byl4jo68lo5/v1 and 10.17504/protocols.io.j8nlkwxxwl5r/v1). Neonate macaque pool experiments (RM-001 to RM-004) were performed at the CNPRC at UC Davis and approved by the local IACUC. Adult macaque pool experiments (RMN-001 and RMN-002) were performed at the NIMH and approved by their local IACUC. Macaques were perfused with ice-cold RNase-free PBS. At the time of perfusion, one hemisphere of the brain was flash frozen and the other hemisphere was sectioned into 4 mm coronal blocks and post-fixed in 4% PFA for 48 h and transferred to Caltech for further processing. For HA staining, we incubated slices with rabbit anti-HA (1:200; Cell Signaling Technology, cat # 3724; RRID: AB_1549585), performed three to five washes with PBS, incubated with donkey anti-rabbit IgG (1:200; Jackson ImmunoResearch Labs, cat # 711-605-152; RRID: AB_2492288) and washed three to five times before mounting. We diluted all the antibodies and performed all the incubations using PBS supplemented with 0.1% Triton X-100 (Sigma-Aldrich, T8787) and 10% normal donkey serum (Jackson ImmunoResearch Labs, cat # 017-000-121; RRID: AB_2337258) overnight at room temperature with shaking.

To isolate the viral DNA and whole RNA, 100 mg slices from the brain and liver were homogenized in TRIzol (Life Technologies, 15596) using a BeadBug (Benchmark Scientific, D1036) and the total DNA and RNA were recovered according to the manufacturer’s recommended protocol. The recovered DNA was treated with RNase, restriction digested with SmaI and purified with a Zymo DNA Clean and Concentrator kit (D4033). The recovered RNA was treated with DNase, and cDNA was generated from the mRNA using SuperScript III (Thermo Fisher Scientific, 18080093) and oligo(dT) primers according to the manufacturer’s recommended protocol. We used PCR to amplify the barcode region using 50 ng of viral DNA or cDNA as the template. After Zymo DNA purification, we diluted the samples at 1:100 and further amplified the barcode region using primers to append adaptors for Illumina NGS. After cleanup, these products were further amplified using NEBNext Dual Index Primers for Illumina sequencing (New England Biolabs, E7600) for ten cycles. We then gel purified the final PCR products using a 2% low-melting-point agarose gel (Thermo Fisher Scientific, 16520050). Pool testing enrichment was calculated identically to library enrichment, but is represented in Fig. 2b,c on a linear scale.

#### Individual characterization of CAP-Mac in rhesus macaques

The details of the procedures in this section can be found on protocols.io (10.17504/protocols.io.5qpvormddv4o/v1 and 10.17504/protocols.io.j8nlkwxxwl5r/v1). Neonate macaques were perfused with PBS and 4% PFA. The brain was sectioned into 4 mm coronal blocks and all the tissue was post-fixed in 4% PFA for 3 days before storage in PBS. The single adult macaque used for in vivo, individual characterization (RM-020; 17 years old, 11 kg) was perfused with RNase-free PBS, and one half-hemisphere was flash-frozen and the other sectioned into 4 mm coronal blocks and post-fixed in 4% PFA. All the tissue was transferred to Caltech for further processing. Brains and livers were sectioned into 100 μm slices using a vibratome. Additionally, sections of brain and spinal cord were incubated in 30% sucrose overnight and embedded in O.C.T. compound (Scigen, 4586) and sectioned into 50 μm slices using a cryostat (Leica Biosystems, CM1950). All the slices were mounted using Prolong Diamond Antifade and imaged using a confocal microscope. For GFP staining of the spinal cord and brain slices from the intrathecally administered macaque, we incubated slices with chicken anti-GFP (1:500; Aves Labs, cat # GFP-1020; RRID: AB_10000240), performed three to five washes with PBS, incubated with donkey anti-chicken IgY (1:200; Jackson ImmunoResearch Lab, cat # 703-605-155; RRID: AB_2340379) and washed three to five times before mounting. We diluted all the antibodies and performed all the incubations using PBS supplemented with 0.1% Triton X-100 (Sigma-Aldrich, T8787) and 10% normal donkey serum (Jackson ImmunoResearch, 017-000-121) overnight at room temperature with shaking.

For morphological reconstruction, we sectioned brains into 300 μm sections and incubated them in a refractive index matching solution^72^ for 72 h before mounting on a slide immersed in the refractive index matching solution. We imaged using a confocal microscope and ×25 objective (LD LCI Plan-Apochromat ×25/0.8 Imm Corr DIC) using 100% glycerol as the immersion fluid. We captured tiled *Z* stacks (1,024 × 1,024 for each frame using the suggested capture settings) around cells of interest and cropped appropriate fields of view for tracing. Tracing was done in Imaris (Oxford Instruments; RRID: SCR_007370) using semi-automated and automated methods.

For neuron (NeuN) and astrocyte (S100β) quantification, the slices were stained using anti-NeuN (EPR12763) antibody (1:200; Abcam, cat # ab177487; RRID: AB_2532109) or anti-S100β antibody (1:200; Abcam, cat # ab52642; RRID: AB_882426) overnight in PBS supplemented with 0.1% Triton X-100 and 10% normal donkey serum. The slices were washed three to five times with PBS and incubated overnight in anti-rabbit IgG antibody conjugated with Alexa Fluor 647 (1:200; Jackson ImmunoResearch Labs, cat # 711-605-152; RRID: AB_2492288) in PBS + 0.1% Triton X-100 + 10% normal donkey serum. After three to five washes and mounting using Prolong Diamond Antifade, we obtained *Z* stacks using a confocal microscope and a ×25 objective. We segmented NeuN- and XFP-positive cells using custom scripts in Python (RRID: SCR_008394) and Cellpose (https://www.cellpose.org/; RRID: SCR_021716)^73^.

#### Ex vivo two-photon imaging

Brain slices of sizes suitable for imaging were prepared with a thickness of 400 µm from larger slices using a vibratome and stored in artificial cerebrospinal fluid bubbled with carbogen gas before two-photon imaging, as previously described in published protocols^74,75^. For testing GCaMP8s responses, electrical stimulation (4–5 V, 80 Hz, 0.3 s duration) with the indicated number of pulses was delivered using an extracellular monopolar electrode placed 100–200 µm away from the neuron imaged. The frame rate of imaging was 30 Hz. Traces of segmented regions of interest were plotted as Δ*F*/*F*_0_ = (*F*(*t*) − *F*_0_)/*F*_0_, where *F*_0_ is defined as the average of all the fluorescence values before the electrical stimulation. The rise time was defined as the time required for the rising phase of the signal to reach from 10% of the peak to 90% of the peak. The decay time constant was obtained by fitting the sums of exponentials to the decay phase of the signal. The signal-to-noise ratio was obtained by dividing the peak amplitude of the signal by the standard deviation of the fluorescence trace before the electrical stimulation.

#### Characterization in adult rhesus macaque slice

One adult rhesus macaque (14 years and 1 month old; 10.83 kg) from the Washington National Primate Research Center was planned for routine euthanasia, and the brain was collected as part of the facility’s Tissue Distribution Program. A block of the superior temporal gyrus was sectioned into 300 μm slices and the slices were recovered^74^ and cultured on an air–liquid membrane interface, as previously described^76^. Approximately 30 min after plating the slices, we administered 1–2 μl of AAV (5 × 10^13^ vg ml^−^^1^ of AAV9 or AAV.CAP-Mac packaging either ssCAG-FXN-HA or ssCAG-eGFP). The experiments were performed in biological triplicates for each condition and the culture medium was refreshed every 48 h until tissue collection at 8 days post-transduction. On the day of tissue collection, the slices were imaged to confirm transduction, slices were cut in half and each half slice was flash frozen in a dry ice–ethanol bath. The samples were stored at −20 °C until further processing.

Each half slice was processed (one each for DNA and RNA recovery). DNA was isolated using the Qiagen DNeasy Blood and Tissue kit (Qiagen, catalogue # 69504) and RNA was recovered using TRIzol (Thermo Fisher Scientific, catalogue # 15596026) and the PureLink RNA Mini kit (Thermo Fisher Scientific, catalogue # 12183018A). DNA was removed from the RNA sample by modifying the first wash of the PureLink RNA Mini kit as follows: wash with 350 µl of Wash Buffer 1, then add 80 µl of RNase-free DNaseI in RDD buffer (Qiagen catalogue # 79254) and incubate the column at room temperature for 15 min; then, wash again with 350 µl of Wash Buffer 1 before proceeding with the protocol. We performed first-strand cDNA synthesis from 400 ng total RNA in 20 µl reactions using a Promega GoScript Reverse Transcription kit (Promega, catalogue # A5000).

We then evaluated the vector genomes and viral transcripts found in each sample using quantitative PCR on a Roche Lightcycler II. Here 100 ng of DNA was used in a 20 µl amplification reaction using TaqMan probes from Thermo Fisher Scientific (EGFP-FAM probe, assay ID Mr04097229_mr, catalogue #4331182; custom genomic reference probe CN2386-2-VIC, Assay ID ARH6DUK, catalogue #4448512, designed to target both *M. mulatta* and *Macaca nemestrina*).

### Green monkey experiments

All the green monkey (*C. sabaeus*) procedures were performed at Virscio and approved by their IACUC. All the monkeys were screened for neutralizing antibodies and confirmed to have <1:5 titre. At approximately 7–8 months of age (1.0–1.3 kg), the monkeys were intravenously dosed (Supplementary Table 6). Dose formulations were allowed to equilibrate to approximately room temperature for at least 10 min, but no more than 60 min before dosing. The IV dose volumes were based on day 0 body weights. The animals were sedated with ketamine (8.0 mg kg^−^^1^) and xylazine (1.6 mg kg^−^^1^). The injection area was shaved and prepped with chlorohexidine and 70% isopropanol and surgically scrubbed before insertion of the IV catheter. Dosing occurred with a single IV infusion of AAV (7.5 × 10^13^ or 7.6 × 10^13^ vg kg^−^^1^) on day 0 via the saphenous vein administered using a hand-held infusion device at a target rate of 1 ml min^−^^1^. General well being was confirmed twice daily by cage-side observation beginning 1 week before dosing. At the scheduled euthanisia time, the monkeys were sedated with ketamine (8–10 mg kg^−^^1^ intramuscular) and euthanized with sodium pentobarbital (100 mg kg^−^^1^ IV to effect). On loss of corneal reflex, a transcardiac perfusion (left ventricle) was performed with chilled PBS using a peristaltic pump set at a rate of approximately 100 ml min^−^^1^ until the escaping fluid ran clear before tissue collection. Cubes of tissue were collected from the left brain hemisphere and various other organs and frozen in the vapour phase of liquid nitrogen for further processing for biodistribution. The right brain hemisphere was removed and cut into ~4 mm coronal slices and post-fixed intact with approximately 20 volumes of 10% neutral-buffered formalin for approximately 24 h at room temperature.

Genomic DNA was extracted from CNS and peripheral tissues using the Thermo Fisher MagMax DNA Ultra 2.0 extraction kit (catalogue # A36570). DNA was assessed for yield by fluorometric quantification with the Qubit dsDNA assay. Approximately 20 ng of DNA was loaded into each 20 μl reaction and the plates were run on the BioRad CFX Connect Real-Time PCR Detection System (catalogue # 1855201). The viral copy number assay was validated for specificity by the detection of a single amplified product; sensitivity, by assessing the lower limit of detection to be greater than ten copies per reaction; and linearity, by ensuring the standard curve *R*^2^ was >0.95. Reactions were assembled in FastStart Universal SYBR Green Master (Rox) (catalogue catalogue # 4913850001). The sequences of the primers were ACGACTTCTTCAAGTCCGCC (forward) and TCTTGTAGTTGCCGTCGTCC (reverse). The PCR protocol used an initial denaturation step of 95 °C for 180 s, followed by 40 cycles of 95 °C for 15 s and 60 °C for 60 s, with an imaging step following each 60 °C cycle. A standard curve was generated with linearized plasmid containing the GFP template sequence present in the virus from 1 × 10^8^ to 1 × 10^10^ copies, diluted in naïve untreated macaque DNA samples prepared using an identical kit as the samples in this study to control for matrix effects. Copies of viral DNA were calculated from the standard curve using the equation for the line of the best fit. Multiplicity of infection values were calculated based on the measured total genomic weight of the host cell DNA per reaction.

Post fixation, the tissues were placed into 10% > 20% > 30% sucrose for 24 h each at 4 °C and then embedded in O.C.T. compound and stored at −80 °C until cryosectioning. The tissue blocks were brought up to −20 °C in a cryostat before sectioning into 30 μm slices and dry mounted onto slides after cryosectioning. After sectioning, the slides were left at room temperature overnight to dry. To assist in neuron quantification, we stained sections with the following antibodies and concentrations: rabbit anti-GFP (1:100; Millipore-Sigma, cat # AB3080; RRID: AB_91337) and mouse anti-NeuN (A60) (1:500; Millipore-Sigma, cat # MAB377; RRID: AB_2298772). For secondary antibody staining, the following secondary antibodies and concentrations were used: donkey anti-rabbit Alexa Fluor 488 (1:500; Thermo Fisher Scientific, cat # A-21206; RRID: AB_2535792) and donkey anti-mouse Alexa Fluor 647 (1:500; Thermo Fisher Scientific, cat # A-31571; RRID: AB_162542). All the antibodies were diluted with 1× PBS supplemented with 0.25% Triton X-100 (PBST) and 5.00% normal donkey serum. Primary antibody incubations were left overnight at room temperature. Sections were then washed with PBST. Secondary antibody incubations were performed for 2 h at room temperature. The sections were washed thrice in PBST. The sections were incubated in DAPI solution (1:10,000; Invitrogen, D1306) at room temperature for 5 min and then washed. Sections were coverslipped using Prolong Diamond Antifade.

Three sections per animal were stained and imaged. Each section was imaged in triplicate, with each region of interest having a total of nine images. Tissue regions of interest were imaged with a Keyence BZ-X800 with the following acquisition parameters: GFP (1/500 s), Cy5 (1 s), DAPI (1/12 s), high-resolution *Z* stack at 1.2 µm pitch. The following brain subregions were imaged: frontal, parietal, temporal, occipital cortices, cerebellum, caudate, putamen and thalamus (medial, ventral lateral and ventral posterior nuclei). A semi-automated cell-counting method was performed using ImageJ (RRID: SCR_003070) for quantification. Using thresholds and particle analysis, we quantified NeuN-positive and DAPI-positive cells. Using ImageJ’s cell counter, we manually counted GFP-positive cells as well as GFP and NeuN double-positive cells.

### iPSC experiments

Neuronal cultures were produced by differentiating and maturing iPSC-derived neural progenitor cells with Stemdiff Forebrain Differentiation and Maturation kits (StemCell # 08600 and # 08605, respectively), according to their manufacturer’s protocols. Neural progenitor cells were produced by differentiation of the foreskin fibroblast-derived iPSC line: ACS-1019 (ATCC# DYS-0100; RRID: CVCL_X499), with Stemdiff SMADi Neural Induction kits (StemCell l#08581), selection with Stemdiff Neural Rosette Selection Reagent (StemCell l#05832) and expansion in Stemdiff Neural Progenitor Media (StemCell l#05833), according to their manufacturer’s protocols. Neurons were matured a minimum of 8 days before replating for transduction.

Mature neuronal cultures, seeded 15,000 cells per well in polyornithine- and laminin-coated black-walled 96-well optical plates, were cultured for an additional 4 days before transduction. Replicate wells were transduced with virus serially diluted across six orders of magnitude in 90% maturation media and 10% OptiPRO SFM. Four days post-transduction, the cultures were fixed with 4% PFA and counterstained with 1 µg ml^−^^1^ Hoechst 33322. The identification of transduced cells was determined by imaging 60 fields per well, using two-channel fluorescence detection (Hoechst, ex386/em440; eGFP, ex485/em521) on a CellInsight CX5 HCS Platform. Individual cells were identified by Hoechst detection of their nuclei and applying size- and contact-constrained ring masks to each cell. Cell transduction was determined by measuring the eGFP fluorescence above a threshold level within an individual ring mask. For each population, the percentage of transduced cells was plotted versus the applied dose. Curve fits and EC_50_ values were determined with Prism GraphPad (RRID: SCR_002798) agonist versus response (three-parameter) regression method. To report per-cell eGFP expression efficiencies, the eGFP spot fluorescence intensities were averaged from each ring mask across a minimum of 5,000 cells per well. The curve fits were obtained using the Prism GraphPad Biphasic X as the concentration regression method.

### Statistics and reproducibility

For the representative images, at least three separate slices from each sample were mounted for imaging. Within each brain region of a single animal, at least three different fields of view were taken (minimum field of view after tiling, 2.38 mm × 2.38 mm; slice thickness, 50 µm), equating to nine separate fields of view across three brain slices, to ensure consistency across imaging samples.

### Reporting summary

Further information on research design is available in the Nature Portfolio Reporting Summary linked to this article.

## Online content

Any methods, additional references, Nature Portfolio reporting summaries, source data, extended data, supplementary information, acknowledgements, peer review information; details of author contributions and competing interests; and statements of data and code availability are available at 10.1038/s41565-023-01419-x.

### Supplementary information

Supplementary Information Supplementary Fig. 1 and Tables 1–6.

Reporting Summary

### Source data

Source Data Fig. 2 Statistical source data.

Source Data Fig. 3 Statistical source data.

Source Data Fig. 5 Statistical source data.

Source Data Fig. 6 Statistical source data.

Source Data Extended Data Fig. 2 Statistical source data.

Source Data Extended Data Fig. 3 Statistical source data.

Source Data Extended Data Fig. 6 Statistical source data.

Source Data Extended Data Fig. 7 Statistical source data.

## Data Availability

The capsid plasmid used to produce AAV.CAP-Mac is available on Addgene (200658; RRID: Addgene_200658). Imaging datasets are available on the Brain Imaging Library (10.35077/g.948). Source data are available for Figs. 2, 3, 5 and 6 and Extended Data Figs. 2, 3, 6 and 7. All other data that support the findings of this study are available from the corresponding authors upon reasonable request. Source data are provided with this paper.

## Code availability

NeuN quantification was performed using CellPose (https://www.cellpose.org/; RRID: SCR_021716)^73^ and ImageJ (RRID: SCR_003070). All NGS data were processed using previously published open-source software from our lab (https://github.com/GradinaruLab/protfarm and https://github.com/GradinaruLab/mCREATE)^16^. All code specifically associated with this manuscript is available via GitHub at https://github.com/GradinaruLab/CAP-Mac (10.5281/zenodo.8040857).
