# Supplementary material for: Adeno-associated viral vectors for functional intravenous gene transfer throughout the non-human primate brain
Source: Nat Nanotechnol. Author manuscript; Available in PMC 2023 Oct 23. (PMC10575780; doi:10.1038/s41565-023-01419-x)
Supplement: Supplementary Info [file NIHMS1920098-supplement-Supplementary_Info.pdf]

Supplementary Fig. 1: Generating AAV libraries and choosing variants for further characterization.

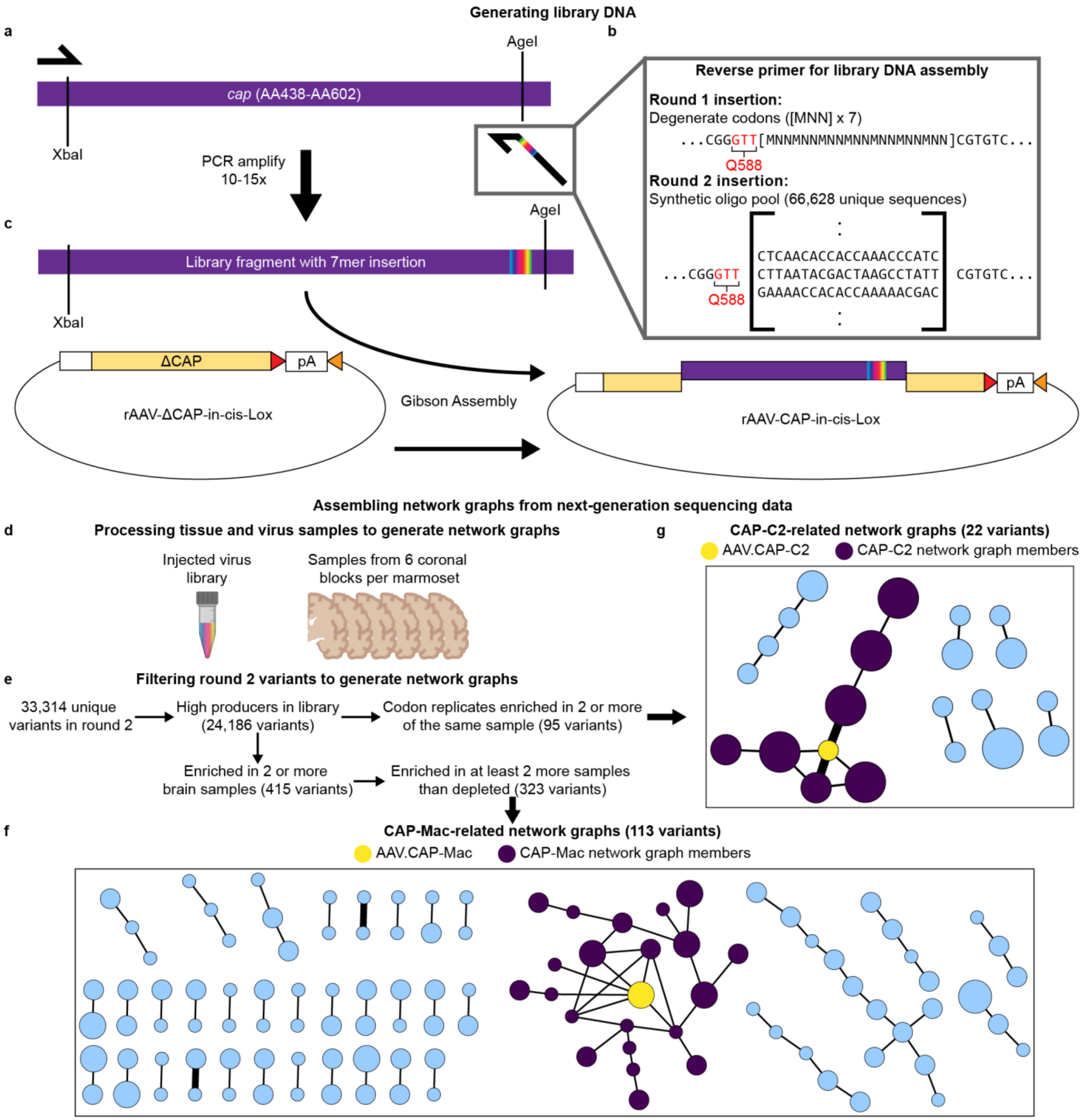

Supplementary Fig. 1: Generating AAV libraries and choosing variants for further characterization. a, We introduced diversity into the AAV9 *cap* genome using a reverse primer with a 21-nucleotide insertion after Q588. The reverse primer was used to generate a PCR fragment approximately spanning the XbaI to AgeI sites of the modified *cap* gene (approximately AA438 to AA602). **b**, For DNA assembly for round 1 selections, the reverse primer contained 7 degenerate codons ([MNN] x 7]). For round 2 selections, we used a synthetic oligo pool to specify each 21 bp sequence that we inserted into *cap*. **c**, The PCR-amplified fragment contained homologous regions that overlap with the rAAV-

ΔCAP-in-cis-Lox digested plasmid, and the two fragments were assembled using Gibson assembly to create the final assembled library DNA. **d-g**, Process to assemble network graphs from next-generation sequencing data. While our previous CREATE-based selections have relied on Cre-transgenic mouse lines to increase selection stringency, Cre-transgenic marmosets are currently unavailable, and we were unable to confer this additional selective pressure during selections. We reasoned that through this clustering analysis, we could efficiently and productively sample variants from our selections to (1) limit the number of animals used for individual characterization and (2) partially overcome the absence of the selective pressure provided by Cre-transgenic mice in CREATE. **d**, To generate network graphs, we processed the injected virus library and sampled from each of the 6 brain sections from each animal. **e**, From our next-generation sequencing data, we calculated library enrichment scores and filtered the variants using two separate criteria. **f**, **g**, Network graphs for AAV.CAP-Mac (**f**) and AAV.CAP-C2 (**g**). CAP-Mac and CAP-C2 were both chosen because they were the most interconnected nodes within their respective networks. Each node represents a unique variant recovered from the round 2 selection and each edge represents pairwise reverse Hamming distance ≥ 3.

Supplementary Table 1: Summary of CAP-Mac cellular tropism in brain of multiple species.

Species	Strain or colony of origin	Developmental stage	Route of administration	Cellular tropism
Mus musculus	C57BL/6J (The Jackson Laboratory)	P0	Intravenous (temporal vein)	Non-specific (astrocytes, neurons, vasculature)
Mus musculus	C57BL/6J, BALB/cJ, DBA/2J (The Jackson Laboratory)	Adult (6-8 weeks)	Intravenous (retro-orbital sinus)	Vasculature
Mus musculus	C57BL/6J, BALB/cJ, DBA/2J (The Jackson Laboratory)	Adult (6-8 weeks)	Intracerebroventricular (lateral ventricle)	Neuronal
Callithrix jacchus	UC San Diego (La Jolla, CA)	Adult	Intravenous (saphenous vein)	Vasculature
Macaca mulatta	California National Primate Research Center (Davis, CA)	Infant	Intravenous (saphenous vein)	Neuronal
Macaca mulatta	California National Primate Research Center (Davis, CA)	Infant	Intra-cisterna magna	Neuronal
Chlorocebus sabaeus	Virscio (St. Kitts)	Infant	Intravenous (saphenous vein)	Neuronal
Macaca mulatta	California National Primate Research Center (Davis, CA)	Adult	Intravenous (saphenous vein)	Astrocyte-biased

Supplementary Table 2: Assessment of tissue by an independent pathologist.

Animal ID	Capsid	Cargo	Route of administration	Tissue	Degeneration score	Inflammation score
RM-001	8 capsid pool*	CAG-FXN-HA-Barcode	Intravenous	Brain	1	-
RM-002	8 capsid pool*	CAG-FXN-HA-Barcode	Intravenous	Brain	0	1
RM-009	AAV.CAP-Mac	CAG-mNeonGreen, CAG-mRuby2, CAG-mTurquoise2 cocktail	Intravenous	Brain	0	0
				Liver	0	1
				Kidney	0	0
				Spinal cord & dorsal root ganglia	0	0
RM-010	AAV.CAP-Mac	CAG-mNeonGreen, CAG-mRuby2, CAG-mTurquoise2 cocktail	Intravenous	Brain	1	1
RM-010				Liver	0	1
RM-010				Kidney	0	0
RM-010				Spinal cord & dorsal root ganglia	1	1

**8 capsid pool consisted of AAV9, PHP.eB, 452sub-LUNG1, CAP-B2, CAP-B10, CAP-B22, CAP-Mac, CAP-C2.*
Degeneration and inflammation were scored on a scale from 0 (no observed pathology) to 5.

Supplementary Table 3: Marmoset information for dual-injection characterization of CAP-Mac.

Marmoset ID	Age (years)	Sex	Route of administration	Capsid*	Cargo*	Weight at injection (kg)	Total dose (vg/kg)	Expression length (days)
Sandy	5.8	Female	Intravenous	(1) AAV9<br>(2) AAV.CAP-Mac	(1) CAG-eGFP<br>(2) CAG-mRuby3	0.468	2×10^{13}	31
Conan	2.8	Male	Intravenous	(1) AAV.CAP-Mac<br>(2) AAV9	(1) CAG-eGFP<br>(2) CAG-mRuby3	0.386	2×10^{13}	31

All experiments were performed in the Cortical Systems and Behavior Laboratory at UCSD.
**Numbers in "Capsid" and "Cargo" column correspond to capsid-cargo pairs for each dual injection.*

Supplementary Table 4: Rhesus macaque information for variant pool testing.

Macaque ID	Age	Sex	Route of administration	Weight at injection (kg)	Total dose (vg/kg)	Expression length (days)
RM-001	1 day	Male	Intravenous	0.602	1×10^{14}	31
RM-002	1 day	Male	Intravenous	0.454	1×10^{14}	31
RM-003	150 days	Female	Intra-cisterna magna	1.407	1.5×10^{12}	55
RM-004	150 days	Female	Intra-cisterna magna	1.307	1.5×10^{12}	55
RMN-001	7.9 years	Male	Intravenous	5.3	7.5×10^{13}	40
RMN-002	7.8 years	Female	Intravenous	4.65	7.5×10^{13}	41

RMN-001 and RMN-002 experiments were performed at the NIMH. All other experiments were performed at the California National Primate Research Center at UC Davis. All macaques were injected with pool of 8 variants (AAV9, PHP.eB, 452sub-LUNG1, CAP-B2, CAP-B10, CAP-B22, CAP-Mac, CAP-C2) packaging CAG-hFXN-HA-Barcode.

Supplementary Table 5: Rhesus macaque information for characterization of CAP-Mac.

Macaque ID	Age	Sex	Route of administration	Capsid*	Cargo*	Weight at injection (kg)	Total dose (vg/kg)	Expression length (days)
RM-008	29 days	Male	Intra-cisterna magna	AAV.CAP-Mac	CAG-GCaMP7s	0.74	2.5×10^{13}	68
RM-009	2 days	Male	Intravenous	AAV.CAP-Mac	CAG-mNeonGreen, CAG-mRuby2, CAG-mTurquoise2 cocktail	0.956	5×10^{13}	29
RM-010	2 days	Male	Intravenous	AAV.CAP-Mac	CAG-mNeonGreen, CAG-mRuby2, CAG-mTurquoise2 cocktail	0.522	5×10^{13}	77
RM-015	3 days	Male	Intravenous	AAV.CAP-Mac	CAG-GCaMP8s and hSyn-hM3D(Gq)-mCherry cocktail	0.635	4×10^{13}	34
RM-017	1 day	Female	Intravenous	AAV.CAP-Mac	CAG-GCaMP8s and CAG-ChRmine-TS-mRuby3 cocktail	0.46	4×10^{13}	43
RM-020	17.2 years	Female	Intravenous	(1) AAV.CAP-Mac (2) AAV9	(1) CAG-eGFP (2) CAG-mRuby3	11.04	2×10^{13}	43

*All experiments were performed at the California National Primate Research Center at UC Davis. *Numbers in "Capsid" and "Cargo" column correspond to capsid-cargo pairs for each dual injection.*

Supplementary Table 6: Green monkey information for individual characterization of CAP-Mac.

Green monkey ID	Age (days)	Sex	Route of administration	Capsid	Cargo	Weight at injection (kg)	Total dose (vg/kg)	Expression length (days)
C010	200	Male	Intravenous	AAV.CAP-MAC	CAG-EGFP	1.18	7.5×10^{13}	36
C017	172	Male	Intravenous	AAV.CAP-MAC	CAG-EGFP	1.32	7.6×10^{13}	36
C002	215	Male	Intravenous	AAV9	CAG-EGFP	1.31	7.5×10^{13}	36
C016	178	Male	Intravenous	AAV9	CAG-EGFP	1.02	7.6×10^{13}	37

All experiments were performed at Virscio, Inc.
