# Supplementary material for: Adeno-associated viral vectors for functional intravenous gene transfer throughout the non-human primate brain
Source: Nat Nanotechnol. 2023 Jul 10;18(10):1241–51. doi: 10.1038/s41565-023-01419-x (PMC10575780; doi:10.1038/s41565-023-01419-x)
Supplement: Supplementary file 2 [file 41565_2023_1419_MOESM2_ESM.pdf]

nature research

Corresponding author(s): Viviana Gradinaru

Last updated by author(s): April 3, 2023

Reporting Summary

Nature Research wishes to improve the reproducibility of the work that we publish. This form provides structure for consistency and transparency in reporting. For further information on Nature Research policies, see our Editorial Policies and the Editorial Policy Checklist.

Statistics

For all statistical analyses, confirm that the following items are present in the figure legend, table legend, main text, or Methods section.

n/a	Confirmed	
☐	☒	The exact sample size (*n*) for each experimental group/condition, given as a discrete number and unit of measurement
☐	☒	A statement on whether measurements were taken from distinct samples or whether the same sample was measured repeatedly
☐	☒	The statistical test(s) used AND whether they are one- or two-sided<br>*Only common tests should be described solely by name; describe more complex techniques in the Methods section.*
☐	☒	A description of all covariates tested
☐	☒	A description of any assumptions or corrections, such as tests of normality and adjustment for multiple comparisons
☐	☒	A full description of the statistical parameters including central tendency (e.g. means) or other basic estimates (e.g. regression coefficient) AND variation (e.g. standard deviation) or associated estimates of uncertainty (e.g. confidence intervals)
☐	☒	For null hypothesis testing, the test statistic (e.g. *F*, *t*, *r*) with confidence intervals, effect sizes, degrees of freedom and *P* value noted<br>*Give P values as exact values whenever suitable.*
☒	☐	For Bayesian analysis, information on the choice of priors and Markov chain Monte Carlo settings
☒	☐	For hierarchical and complex designs, identification of the appropriate level for tests and full reporting of outcomes
☒	☐	Estimates of effect sizes (e.g. Cohen's *d*, Pearson's *r*), indicating how they were calculated

Our web collection on statistics for biologists contains articles on many of the points above.

Software and code

Policy information about availability of computer code

Data collection	Confocal images were obtained using ZEN (Black edition).
Data analysis	Confocal images were processed using ZEN (Blue edition). NeuN and S100-beta quantification was obtained using CellPose and ImageJ (version 1.53n_7). All next-generation sequencing data was processed using previously published open-source software from our lab (https://github.com/GradinaruLab/protfarm and https://github.com/GradinaruLab/mCREATE). Clustering analysis was visualized using Cytoscape (ver. 3.9.0). All figures and statistical testing was completed using Python. All code and data associated with this manuscript can be found at https://github.com/GradinaruLab/CAP-Mac

For manuscripts utilizing custom algorithms or software that are central to the research but not yet described in published literature, software must be made available to editors and reviewers. We strongly encourage code deposition in a community repository (e.g. GitHub). See the Nature Research guidelines for submitting code & software for further information.

Data

Policy information about availability of data

All manuscripts must include a data availability statement. This statement should provide the following information, where applicable:

- Accession codes, unique identifiers, or web links for publicly available datasets
- A list of figures that have associated raw data
- A description of any restrictions on data availability

The capsid plasmid used to generate AAV.CAP-Mac is available on Addgene (200658). Source data is available for Figures 2, 3, 5, and 6 and Extended Data Figures 2, 3, 6, and 7. All other data that support the findings of this study are available from the corresponding author upon reasonable request.

Field-specific reporting

Please select the one below that is the best fit for your research. If you are not sure, read the appropriate sections before making your selection.

☒ Life sciences ☐ Behavioural & social sciences ☐ Ecological, evolutionary & environmental sciences

For a reference copy of the document with all sections, see nature.com/documents/nr-reporting-summary-flat.pdf

Life sciences study design

All studies must disclose on these points even when the disclosure is negative.

Sample size	No sample size calculation was performed. Primate sample size was determined based on animal availability, as there are finite primates available during a given birthing season. To the best of our ability, we tried to do experiments in at least duplicate if possible. For stem-cell culture studies, sample sizes were determined based on availability of stem-cell cultures and limited by the size of a 96-well culture plate. For the ex vivo rhesus macaque slice experiments, these were decided based on the availability of cultured slices, as these cultures are obtained from macaques undergoing planned euthanasia.
Data exclusions	No data was excluded from the analyses.
Replication	Experiments were performed in two Old World Primate species during two independent experiments performed at independent sites. Green monkey experiments were managed by Capsida Biotherapeutics and rhesus macaque experiments were managed by UC Davis/CNPRC and Caltech. All attempts at replication were successful.<br><br>Experiments involving mice were replicated twice and were successful. All attempts at replicating stem-cell culture experiments were successful. Because of the limited availability of ex vivo macaque tissue (only to be obtained during routine euthanasia), the ex vivo macaque experiment was only completed once. Experiments involving marmosets and adult macaques were also only completed once due to the limited availability of marmosets.
Randomization	For green monkey experiments, animal subjects were chosen at random to be administered with AAV9 or AAV.CAP-Mac. Green monkeys were randomized according to staff at Virscio, Inc. Note that all green monkeys were sex and age matched. Otherwise, randomization was not relevant for other experiments. Randomization was not relevant for other studies as the data was interpreted in isolation without comparison across experimental groups. This is due to the limited availability of NHPs. When possible, we did try to include an internal control (e.g. AAV9) to circumvent the need for multiple experimental groups.
Blinding	All animal handling was complete by trained veterinary technicians and researchers at the respective sites who were blinded to capsid choice (e.g. AAV9 or AAV.CAP-Mac).

Reporting for specific materials, systems and methods

We require information from authors about some types of materials, experimental systems and methods used in many studies. Here, indicate whether each material, system or method listed is relevant to your study. If you are not sure if a list item applies to your research, read the appropriate section before selecting a response.

Materials & experimental systems

n/a	Involved in the study	
☐	☒	Antibodies
☐	☒	Eukaryotic cell lines
☒	☐	Palaeontology and archaeology
☐	☒	Animals and other organisms
☒	☐	Human research participants
☒	☐	Clinical data
☒	☐	Dual use research of concern

Methods

n/a	Involved in the study	
☒	☐	ChIP-seq
☒	☐	Flow cytometry
☒	☐	MRI-based neuroimaging

Antibodies

Antibodies used	rabbit anti-HA (1:200; Cell Signaling Technology, 3724)<br>rabbit anti-NeuN (EPR12763) antibody (1:200; Abcam, ab177487)<br>rabbit anti-s100beta antibody (1:200; Abcam, ab52642)<br>rabbit anti-GFP (1:100; Millipore-Sigma, AB3080)<br>mouse anti-NeuN (A60) antibody (1:500; Millipore-Sigma, MAB377)<br>donkey anti-rabbit IgG antibody conjugated with Alexa Fluor 647 (1:200; 711-605-152, Jackson ImmunoResearch)<br>donkey anti-rabbit Alexa Fluor 488 (1:500; Invitrogen, A21206)<br>donkey anti-mouse Alexa Fluor 647 (1:500; Invitrogen, A31571)

Validation	All antibodies were independently validated and verified to have no signal in the absence of the primary antibody. Validation from the manufacturer's website:<br><br>rabbit anti-HA (1:200; Cell Signaling Technology, 3724):<br>HA-Tag (C29F4) Rabbit mAb detects exogenously expressed proteins containing the HA epitope tag. The antibody may cross-react with a protein of unknown origin ~100kDa. Species Reactivity: All Species Expected<br><br>rabbit anti-NeuN (EPR12763) antibody (1:200; Abcam, ab177487) :<br>Reacts with: Mouse, Rat, Sheep, Goat, Cat, Dog, Human, Zebrafish, Common marmoset. Predicted to work with: Pig, Cynomolgus monkey<br><br>rabbit anti-S100beta antibody (1:200; Abcam, ab52642):<br>Reacts with: Mouse, Rat, Human. Predicted to work with: Goat, Zebrafish, Macaque monkey<br><br>rabbit anti-GFP (1:100; Millipore-Sigma, AB3080):<br>AB3080 is made against highly-purified native GFP from Aequorea victoria. It is reactive with GFP from both native and recombinant sources. While other commercial antibodies to GFP typically demonstrate strong reactivity with E. coli, the AB3080 GFP antibody has been purified by Protein A chromatography, followed by extensive adsorption against whole E. coli BL21 strain cells, giving them minimal cross-reactivity with E. coli.<br><br>mouse anti-NeuN (A60) antibody (1:500; Millipore-Sigma, MAB377):<br>species reactivity: avian, pig, chicken, human, rat, salamander, ferret, mouse

Eukaryotic cell lines

Policy information about cell lines

Cell line source(s)	HEK293T cells (ATCC, CRL-3216)<br>foreskin fibroblast-derived iPSC line: ACS™-1019 (ATCC# DYS-0100)
Authentication	None of the cell lines used were authenticated.
Mycoplasma contamination	The cell lines were not tested for mycoplasma contamination.
Commonly misidentified lines<br>(See ICLAC register)	No cell lines used in this studies are commonly misidentified.

Animals and other organisms

Policy information about studies involving animals; ARRIVE guidelines recommended for reporting animal research

Laboratory animals	For mice studies, we purchased C57BL/6J (000664), BALB/cJ (000651), and DBA/2J (000671) mice (all males, 6–8 weeks old) from The Jackson Laboratory. Mice were housed on a 13 hour on, 11 hour off light/dark cycle, with ad libitum access to water and food, under standard conditions (71-75 °F, 30-70% humidity). For Green monkey studies, we used all males approximately 6 months old. For rhesus macaque experiments, we used 6 males and 3 females ranging between newborn and 5 months old, 2 adult macaques approximately 8 years old (one male and one female), and one female adult macaque (17.3 years). For marmoset studies, we used one male (2.8 years) and one female (5.8 years).
Wild animals	The study did not involve wild animals.
Field-collected samples	The study did not involve field-collected samples.
Ethics oversight	All experiments and procedures were approved by local regulatory boards and committees and were required to comply with approved protocols. All mouse procedures were performed at Caltech approved by the California Institute of Technology Institutional Animal Care and Use Committee (IACUC). Marmoset and adult macaque procedures took place at the NIH and were approved by the NIH IACUC. Marmoset procedures were also completed at UCSD, and were in compliance and approved by the UCSD IACUC. Infant macaque procedures took place at California National Primate Research Center at UC Davis, and were approved by their local IACUC. Green monkey procedures took place at Virscio, Inc and were approved by their local IACUC.

Note that full information on the approval of the study protocol must also be provided in the manuscript.
